# Deep learning for nanoscience scanning electron microscope image classification

**DOI:** 10.1038/s41598-026-63864-7

**Published:** 2026-08-19

**Authors:** Neama Sayed, Mourad Raafat Mouhamed, A. Ezzat Labib, Mona M. Soliman, Ashraf Darwish

**Affiliations:** 1 Mathematics Department, Faculty of Science, Capital University (Formerly Helwan University), Cairo, Egypt; 2 Faculty of Information and Artificial Intelligence, Cairo University, Cairo, Egypt; 3 Scientific Research School in Egypt (SRGS), Giza, Egypt

**Keywords:** Material science, Scanning electron microscope (SEM), Deep learning, Transfer learning, Imbalanced data, Engineering, Materials science, Mathematics and computing, Nanoscience and technology

## Abstract

Materials science investigates the relationships between a material’s structure, properties, and fabrication processes. Recently, artificial intelligence (AI) and deep learning (DL) techniques have significantly enhanced the ability to analyze complex visual data, particularly in scanning electron microscopy (SEM) images. These approaches enable the detection of subtle morphological patterns that may not be easily identified through manual inspection, facilitating more accurate characterization of nanomaterials. In this study, a multi-class classification framework is proposed for SEM images of nanostructures, categorized into nanowires, fibers, and tips (NFT). To address the issue of class imbalance, the Synthetic Minority Over-sampling Technique (SMOTE) was incorporated using two complementary strategies: pixel-level and feature-level representations. In the pixel-level approach, synthetic samples were generated directly from flattened image data, while in the feature-level approach, deep features were first extracted using a pre-trained ResNet50 model before applying SMOTE. For classification, transfer learning was employed using three convolutional neural network architectures: SqueezeNet, ShuffleNet, and GoogLeNet. In addition, a Multi-Layer Perceptron (MLP) classifier was used for feature-level representations. The experimental results demonstrate that both SMOTE strategies effectively address class imbalance, while the pixel-level approach achieved the highest classification performance. The pixel-level approach achieved a classification accuracy of up to 98.35% using GoogLeNet and ShuffleNet, whereas the feature-level approach achieved an accuracy of 97.44% while offering a computationally efficient alternative. These findings highlight the effectiveness of combining SMOTE with transfer learning for handling imbalanced SEM datasets and illustrate the trade-off between classification performance and computational efficiency.

## Introduction

Materials science is a broad and interdisciplinary field that integrates and extends concepts from physics, chemistry, and engineering to investigate structures and phenomena at the nanoscale. A key objective of this field is establish relationships between a material’s synthesis and processing history and its internal configuration and/or arrangement, whereby its mechanical, thermal, electronic, and optical properties can be inferred. This structure–property relationship spans multiple length scales, from atomic to macro-scale systems, and forms the foundation for the rational design of advanced materials^1^.

Nanomaterials exhibit unique and often superior properties compared to their bulk counterparts, including enhanced mechanical strength, electrical and thermal conductivity, and increased surface reactivity. These properties arise from nanoscale phenomena such as quantum confinement, surface effects, and modified defect behavior^2,3^. Due to these unique properties, nanomaterials have found widespread applications across various fields. They are extensively used in energy storage, flexible electronics, photonics, catalysis, photovoltaics, sensors, drug delivery, and biomedicine^4,5^.

Scanning Electron Microscope (SEM) is one of the most widely used for microstructural characterization in nanomaterials science. It’s a high-precision instrument for surface morphology and structure characterizations of nanomaterials in the range from micro scale order down to sub-nano level. SEM images are generated by scanning a tightly focused beam of electrons across the surface of a sample. When the electrons collide with atoms in a sample, various signals are generated, including secondary electrons (SE), backscattered electrons (BSE), and X-rays that can be collected to produce images displaying topographical information of surface shape changes across samples, material types and distribution^6^. Despite its effectiveness, SEM image analysis is often performed manually, which is time-consuming and subjective. This challenge becomes more significant when distinguishing between nanostructures with highly similar morphologies, such as nanowires, nanotubes, and fibrous structures^7^. Therefore, there is a growing need for automated and objective analysis methods.

Machine Learning (ML) is a subset of artificial intelligence, which proposes the development and application of algorithms that can enable computers to learn from data in order to improve their task-related performance without being directly programmed for each such task. In materials science, ML has been a highly efficient tool for analyzing large and complex high-dimensional data as well as predicting structure–property relations. It is increasingly used throughout the materials development pipeline for property prediction, materials discovery, defect identification, classification, and microstructure analysis^8,9^. Notably, emerging studies have emphasized the increasing impact of ML on expediting the pace of materials innovation through the integration of computation and experiment^10^. Deep learning is a specialized branch of machine learning that uses neural networks with many layers to model complex patterns in data. For image-based characterization specifically, deep learning algorithms such as Convolutional Neural Networks (CNN) have successfully achieved high accuracy and generality for tasks such as segmentation, phase classification, and defect detection^11,12^.

The potency of ML-embedded SEM analysis has been evidenced in recent years through studies. For instance, Yang et al.^13^ proposed a novel deep learning network model for nanowire segmentation with synthetic data augmentation and achieved better results than conventional strategies. Similarly, Liu et al.^14^ applied deep neural networks to automate the analysis of microscopic images, reducing human error and greatly speeding up the analysis process. In a related context, Azad et al.^15^ developed a deep learning-based framework for autonomous morphological fracture analysis of fiber-reinforced composites, demonstrating the effectiveness of morphology-driven learning in materials characterization. Likewise, Perkgoz^16^ utilized convolutional neural networks with transfer learning to identify optical microscopy images of CVD-grown two-dimensional MoS_2_, achieving high classification accuracy and highlighting the potential of transfer learning in microscopy-based material analysis.

Additionally, domain-specific models have shown promise in detecting damage on complex composite materials, as shown by Manujesh and Prajna^17^. Deya et al.^18^ constructed an ensemble of RetinaNet-based classifiers and stacked them with unsupervised denoising to detect different defect types in CD-SEM images, achieving a highly improved precision. Jin et al.^19^ propose a few-shot learning architecture (SEM-CLIP) based on the CLIP architecture for classifying and segmenting nanoscale defects in SEM images with very limited labeled data. Huang et al.^20^ applied vision transformers (ViTs) to classify wafer defect, and they can achieve more than 90% accuracy with less than 15 training samples per class, which exhibits outstanding generalization in the low-data setting. These breakthroughs herald a new epoch of materials characterization, one in which AI complements traditional experimentation and accelerates discovery, increases reproducibility and deepens the understanding of material behavior at the nanoscale.

These efforts will take SEM imaging to the next level by combining it with ML to automatically and objectively analyze nanostructures to uncover hidden structure–function correlations. This integration is now creating a new data-centric paradigm in materials research, based on the synergy between the algorithms that operate within the computer and the data they generate, complemented with high-spatial-resolution experimental data.

This paper presents a deep learning and transfer learning approach to classify an imbalanced data set of SEM images to identify the morphology of materials. The dataset consists of three classes that have similar morphology (nanowires, fibers, and tips (NFT)), which were initially split into 80% for training and 20% for testing. Subsequently, training set was further split into 80% for training and 20% for validation. The training dataset was imbalanced, with more samples assigned to the nanowires class compared to other classes. To address this issue, the Synthetic Minority Over-sampling Technique (SMOTE) was applied exclusively to the training set using two different strategies. In the first strategy, SMOTE was applied at the pixel level by generating synthetic samples directly from flattened image representations, followed by reshaping them back into image format. In the second strategy, SMOTE was applied at the feature level, where high-level feature representations were first extracted using a pre-trained ResNet50 model, and synthetic samples were generated in the feature space without reconstructing images. In both cases, the validation and test sets remained unchanged to ensure fair evaluation. The multiclass classification task was performed using transfer learning based neural networks (CNN) (SqueezeNet, ShuffleNet, and GoogLeNet). In addition, for the feature-level SMOTE approach, a Multi-Layer Perceptron (MLP) classifier was employed to learn from the extracted feature representations. All models were trained using a categorical cross-entropy loss function with softmax activation in the output layer. The optimization process was carried out using stochastic gradient descent (SGD) with a learning rate of 0.01 and a batch size of 64 for CNN models, while the MLP model was optimized using Adam. Early stopping was applied based on validation performance to prevent overfitting. Finally, the models were evaluated using multiple performance metrics to assess their effectiveness in accurately classifying SEM images of nanomaterials.

The main contribution of this work towards enhancing the automated SEM image classification of nanomaterials using deep learning techniques can be summarized as follows:

- This work proposes a dual-strategy framework that integrates the Synthetic Minority Over-sampling Technique (SMOTE) at both the pixel level and feature level, combined with transfer learning, to effectively address class imbalance in SEM datasets.
- A systematic comparative analysis is conducted between pixel-level and feature-level SMOTE, providing insights into how different data representations influence classification performance, robustness, and computational efficiency.
- A comparative evaluation of GoogLeNet, ShuffleNet, and SqueezeNet is conducted under both imbalanced and balanced learning settings, highlighting their strengths, limitations, and trade-offs for SEM image classification.
- Demonstrating strong classification performance on morphologically similar nanostructures while maintaining computational efficiency, reaching 98.35% in the multiclass classification of nanowires, fibers, and tips (NFT), despite the high similarity in their morphological structures.
- The reliability of the proposed framework is further validated through robustness analysis, k-fold cross-validation, statistical significance testing, and detailed performance evaluation using multiple classification metrics.

In “Related work” section, the recent related work is presented, followed by the materials and methods in “Materials and methods” section, Then, the proposed approach, results, and discussion are presented in “The experimental results analysis” section. Finally, the conclusion and future work are presented in “Conclusions” section.

## Related work

Machine learning and deep learning have recently been applied extensively in various fields, such as nanomaterial identification, achieving higher efficiency and accuracy. Recent developments in deep learning architecture offer superior feature extraction and classification capability. There are different models from deep learning, including CNNs, that have been used in literature to analyze and classify the photos of nanomaterials. One of the most successful methods to achieve higher classification accuracy with these models is transfer learning, particularly on small datasets^21,22^.

Modarres et al.^23^ also applied transfer learning in the classification of scanning electron microscope (SEM) images into ten classes of nanostructures, and it reiterates the relevance of CNNs in the field of nanoscience. Kavuran et al. also introduced the SEM-Net model for SEM image classification by using deep feature selection and binary particle swarm optimization and achieved 99.3% accuracy on nanomaterial datasets^24^.

Gutiérrez et al.^25^ used YOLO architectures trained on both real and semi-synthetic SEM images to identify and detect cubical and quasi-spherical nanoparticles. The models performed reliably across unseen data. Barua et al. proposed a DenseNet201-based approach pretrained on ImageNet for SEM image classification. Using 21,272 images across ten classes, they combined deep feature extraction with INCA and SVM, achieving up to 99.53% accuracy^26^.

Qian et al.^27^ introduced a zero-shot classification pipeline by combining the Segment Anything Model (SAM) for unsupervised segmentation with DINOv2 embeddings. The approach demonstrated high-precision shape classification of nanoparticles in SEM images without needing task-specific training. High-throughput applications, Datta et al.^28^ introduced a NSNet-based, morphology analysis-driven deep learning framework that could process SEM and TEM images in real-time with ~ 96% accuracy and 11 FPS.

Wong et al.^29^ investigated the problem of classification of SEM images when only a limited number of samples per class are available. More specifically, the classification of NFT objects, that is, nanowires, fibers, and tips, which share similar geometric features and are typically hard to classify using manual observations. Different data augmentations were applied to improve the performance of the model on the small dataset, such as reflection, translation, scaling, and rotation. The accuracy of identifying the NFT objects, on comparing (estimating) the deep learning algorithms like SqueezeNet, ShuffleNet, GoogLeNet, MobileNet v2, ResNet, and others, was systematically compared by performing validation. Of these, the GoogLeNet model was thus far the best performing model, achieving a validation accuracy of 97.1% with optimal augmentation, in particular, translation and scaling. Table 1 summarizes the most recent state-of-the-art deep learning based models that were used in the literature to classify SEM images, indicating the positive and negative aspects for each model.

**Table 1:** Overview of deep learning architectures and their performance.

Model	Dataset	Accuracy	Pros	Cons
UniRepLKNet (2023)^21^	ImageNet-1 K (general)	88.0%	Universal CNN with large kernel convs; generalizable across domains	Complex design; not specialized for SEM images
EMCNet (Graph Neural Network) (2024)^22^	SEM micrographs (6 classes)	96.8%	Introduced graph-based EMCNet architecture tailored for SEM image classification; captures spatial relationships between features	Limited comparison with other CNN or transformer models; performance on large-scale datasets not validated
CNN (Inception-v3, Inception-v4, ResNet) (2017)^23^	SEM nanostructure dataset	~ 90%	Early work using deep CNNs for SEM; explored multiple architectures	No recent model; lacks augmentation
SEM-Net (2021)^24^	SEM nanomaterials dataset	99.3%	SEM-Net + PSO feature selection	Custom SEM-specific architecture; limited generalization
YOLOv5 (semi-synthetic) (2022)^25^	Real + semi-synthetic SEM (cubical/quasi-spherical NPs)	Not quantified as %	Realistic training data via simulation; detects small particles	No standard accuracy metrics; limited to shape detection
NFSDense201 (2023)^26^	21,272 SEM images	99.53%	DenseNet201 pretrained on ImageNet + patching + INCA + SVM	Requires patching + manual steps; computationally expensive
CNNs (VGG16, EfficientNetV2) (2023)^27^	LM & SEM microalgae images	99%	Strong cross-modality performance	lacks complexity for broader classification benchmarks
NSNet-based (2022)^28^	SEM + TEM images of nanoparticles	~ 96%	Real-time inference; ideal for high-throughput labs	No in-depth analysis of why models perform differently
CNNs (2022)^29^	SEM images of nanomaterials	97.1%	Strong augmentations + fair comparison of CNNs	Did not use a testing dataset; relied solely on accuracy as the evaluation metric, limiting the assessment of model robustness

The proposed approach in this paper is particularly suitable for real-world applications with shallow, lightweight CNN architectures (GoogLeNet, ShuffleNet, SqueezeNet), achieving high classification accuracy (98.35%) at low computational cost, with class balance achieved based on SMOTE. In contrast to many state-of-the-art models, which necessitate large-scale datasets or complex hybrid frameworks (e.g., DenseNet201 + SVM, graph-based networks), the proposed model is simple, fast, and can be particularly challenging in real-time applications in resource-limited settings such as nanoscience labs or portable SEM systems.

## Materials and methods

In the present work, a multiclass classification of Nanomaterial Scanning Electron Microscope (SEM) images for three morphologically resembling categories-nanowires, fibers, and tips (NFT). Since these nanostructures exhibit distinct functional properties, accurate classification is crucial for their electronic, sensor, and biomedical applications. However, manual classification is inherently subjective, time-consuming, and prone to error, particularly given the subtle morphological variations between categories. A significant challenge is due to the imbalanced class distributions among the training data forest cover types. The nanowire class is overrepresented compared to fibers and tips in the NFT subset. The class imbalance causes biased learning, i.e., deep learning models are outwardly skewed towards the presence of the majority class and thus recall and precision for minority classes degrade. Additionally, the sparsity of available labeled data results in a high overfitting risk, which means this is suitable for a transfer learning approach.

**Fig. 1:**
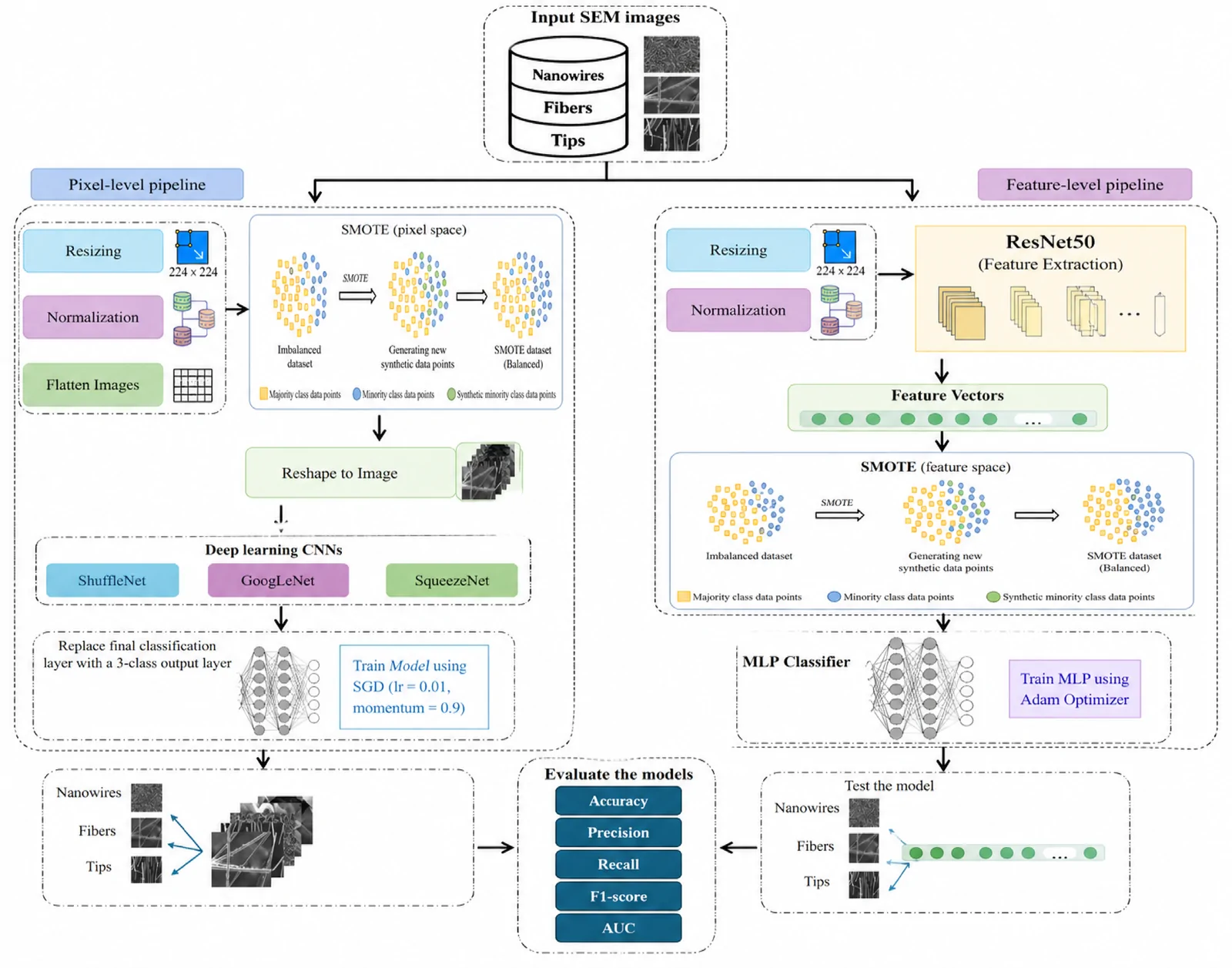
Architecture of the proposed approach.

The central architecture is depicted in Fig. 1 and described in Algorithm 1. The algorithm shows the main steps of the proposed approach, starting with dataset preparation, followed by model training, and finally the evaluation step.

**Algorithm 1:**
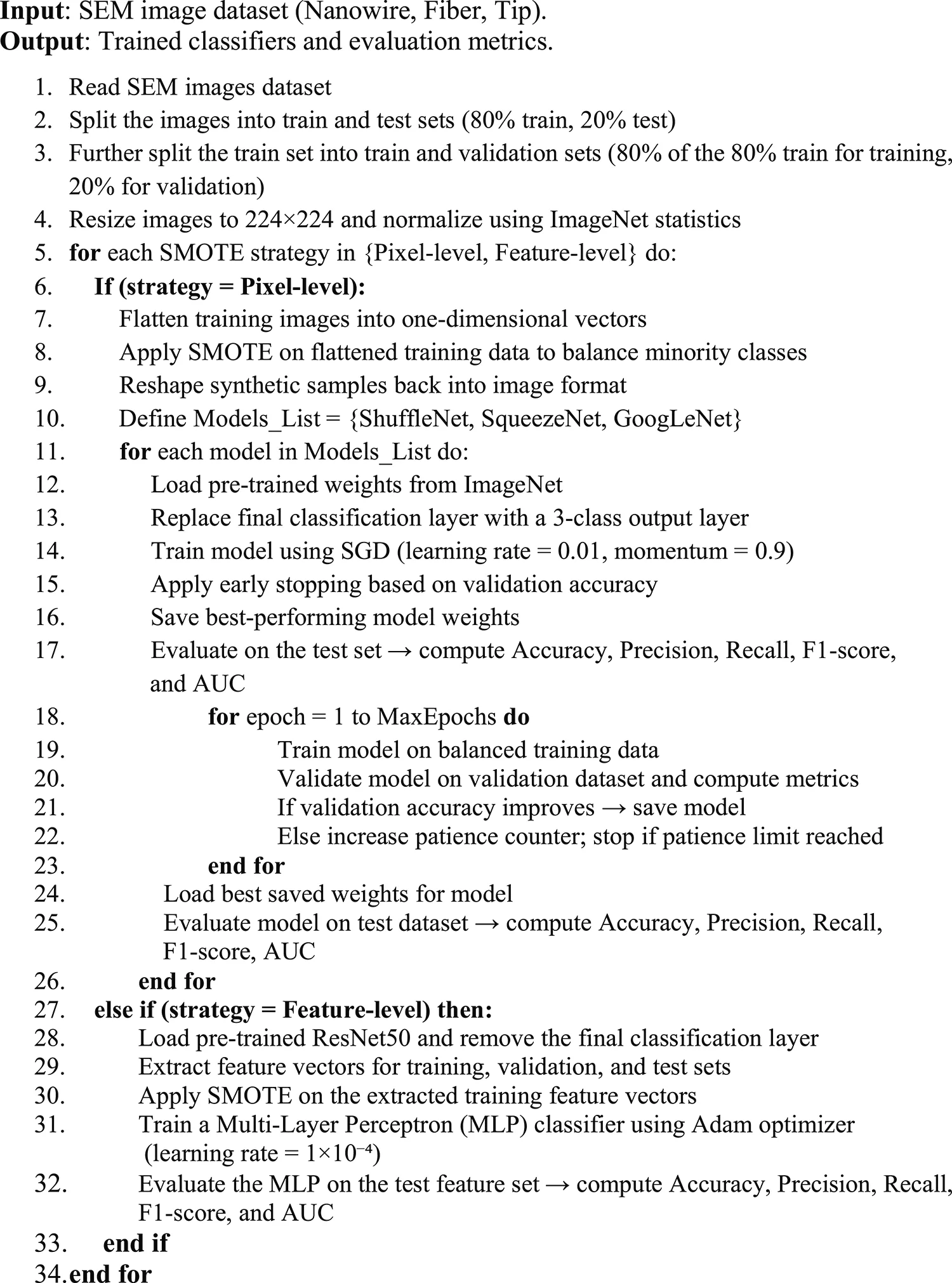
Procedure of multi-classifier classification using transfer learning and SMOTE.

### Dataset

**Fig. 2:**
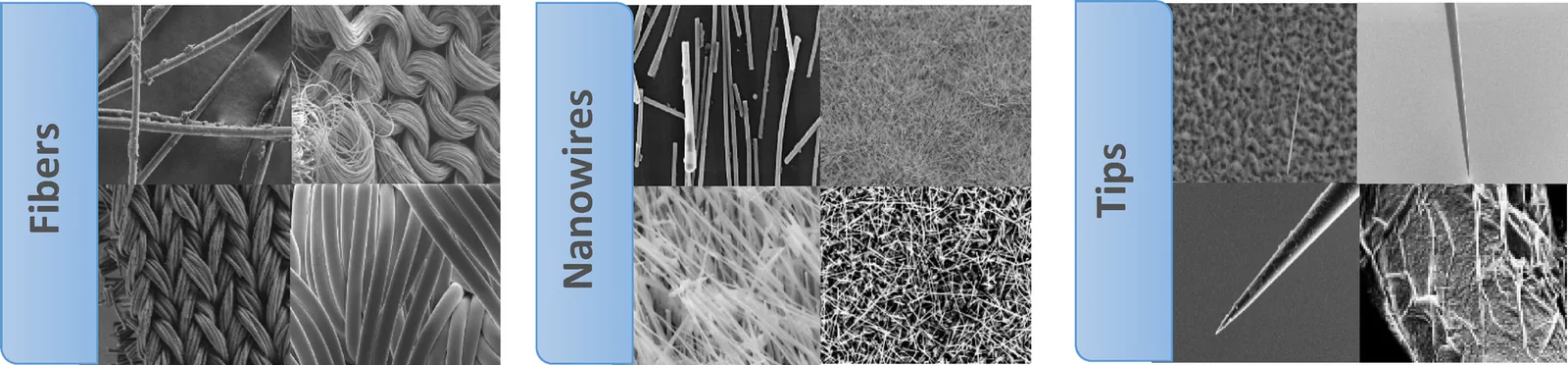
Categories are chosen for the SEM images.

The dataset consists of 18,577 (SEM) images categorized into ten distinct classes: porous sponge, particles, nanowires, fibers, films coated surfaces, powder, patterned surfaces, microelectromechanical system (MEMS) devices, pillars, tips, and biological samples^30^. For this study, particular emphasis is placed on the classification of three morphologically similar categories: nanowires, fibers, and tips. Due to their structural resemblance and subtle differences in geometry, these three categories are collectively referred to as NFT (Nanowire, Fiber, Tip). Representative images for each of the selected NFT categories are illustrated in Fig. 2.

Only the NFT subset was considered in this work. Therefore, images belonging to the Nanowire, Fiber, and Tip classes were first extracted from the original 10-class dataset to form a dedicated three-class dataset. The train, validation, and test splits were subsequently generated using only these selected classes.

To ensure a fair and unbiased evaluation protocol, the NFT dataset was divided into training, validation, and testing subsets before applying any data balancing technique. First, 80% of the images from each class were assigned to the training-validation pool, while the remaining 20% were reserved for testing. Subsequently, 20% of the training-validation pool was allocated to the validation set, resulting in approximately 64%, 16%, and 20% of the data being used for training, validation, and testing, respectively. The distribution of images across the training, validation, and testing subsets before applying SMOTE is presented in Table 2.

**Table 2:** Distribution of SEM images across training, validation, and test sets before applying SMOTE.

Dataset split	Nanowire	Fiber	Tip
Training	2352	103	1039
Validation	588	26	260
Testing	735	33	325
Total	3675	162	1624

To preserve the class distribution across all subsets, the train–validation–test split was performed separately for each class before merging the resulting subsets. This procedure ensured that each class maintained approximately the same proportion in the training, validation, and testing datasets, thereby avoiding additional imbalance introduced during data partitioning.

SMOTE was applied exclusively to the training set, whereas the validation and test sets remained unchanged and contained only real SEM images. This strategy prevents data leakage and ensures that model performance is evaluated on previously unseen real samples.

**Fig. 3:**
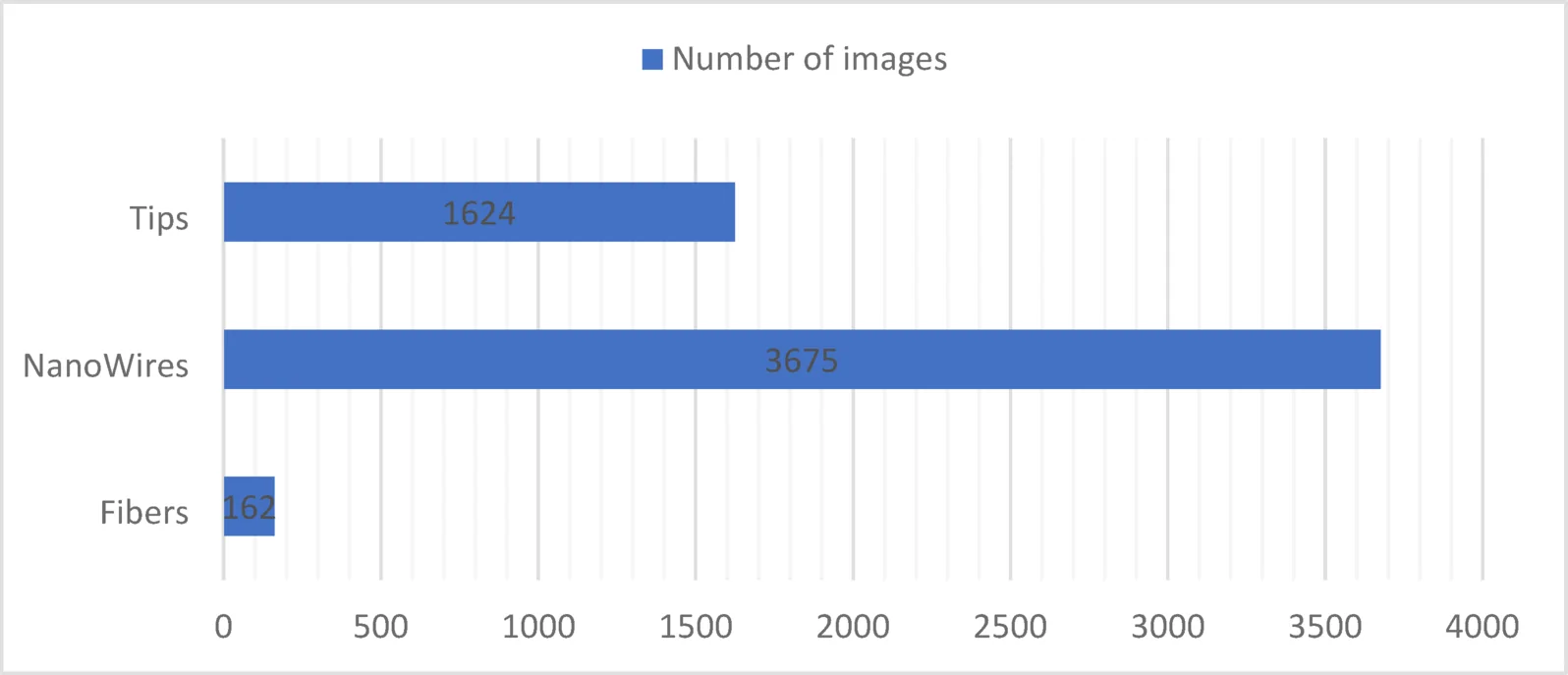
Classes distribution in dataset.

Figure 3 shows the distribution of class samples per category, and one can notice the imbalance in the NFT category. The number of image samples for fibers and tips is particularly limited, compared to nanowire image samples. This imbalance poses problems for learning models since classifiers can get biased towards predicting the majority class. To solve this problem, the Synthetic Minority Over-Sampling Technique (SMOTE) is used to oversample the minority classes and create a more balanced dataset.

### Data preprocessing

In order to make the classification task more effective and uniform, some preprocessing operations were performed on the SEM images.

*Resizing* The raw images were acquired in different sizes, which might influence the training of deep learning algorithms due to input size inconsistency. In order to process these input images based on the deep learning architectures, all input images were resized to a size of 224 × 224 pixels. This resizing process is carried out to facilitate the uniformity of the dataset and also helps in efficient processing by CNNs.

*Normalization* Normalization is an essential preprocessing step in deep learning that helps to improve the model’s performance by standardizing the input data. In this experiment, image pixels values were first scaled to the range ^1^. Then, normalization was applied using predefined values: the mean values [0.485, 0.456, 0.406] and the standard deviation values [0.229, 0.224, 0.225]. These statistics, originally computed on the ImageNet dataset, are widely used in computer vision applications to ensure compatibility with models. This process allows the model to converge much faster and improves its ability to generalize during training.

### SMOTE

*Data balancing* An imbalanced dataset is one in which a particular class (majority) is more than the others, leading machine learning models to favor majority classes and underperform on minority classes. Scientific and industrial datasets like SEM images of nanomaterials face this problem more often, as the minority class is generally much less in number. Techniques to address this issue are divided into three categories: data-level methods such as oversampling and undersampling, algorithm-level methods such as cost-sensitive learning, and hybrid methods combining both^31^.

Among several methods proposed to deal with class imbalance, SMOTE has gained popularity as a simple and effective method that can be applied to different domains. Unlike standard oversampling approaches, which duplicate available minority samples and thus predispose to overfitting, SMOTE produces synthetic instances for minority classes by locating existing samples and generating new instances in their proximity. This method increases minority class diversity and makes the balance of the training set better and more efficient. This allows the model to learn a decision boundary that is more general and robust, which is crucial when working with high-dimensional feature spaces such as SEM image data^32^.

The dataset was initially divided into training, validation, and testing sets. To address class imbalance, SMOTE was applied exclusively to the training set to avoid data leakage. Two different representations of the data were considered for applying SMOTE:

#### Pixel-level SMOTE

In this approach, each image was resized and transformed into a one-dimensional vector by flattening the pixel values. SMOTE was then applied in this high-dimensional feature space to generate synthetic samples for minority classes. The default SMOTE configuration was used with *k* = *5* nearest neighbors, following the implementation provided by the imbalanced-learn library. Nearest neighbors were identified using the Euclidean distance metric implemented in the SMOTE algorithm. After oversampling, the generated vectors were reshaped back into image format and stored for training. The validation and test sets remained unchanged and consisted only of original images. A similar approach has been adopted in prior studies on medical image classification, such as COVID-19 chest X-ray analysis, where SMOTE was applied to flattened image representations to address class imbalance. After generating synthetic samples, the resulting vectors were reshaped back into image format and used for training^33^.

**Fig. 4:**
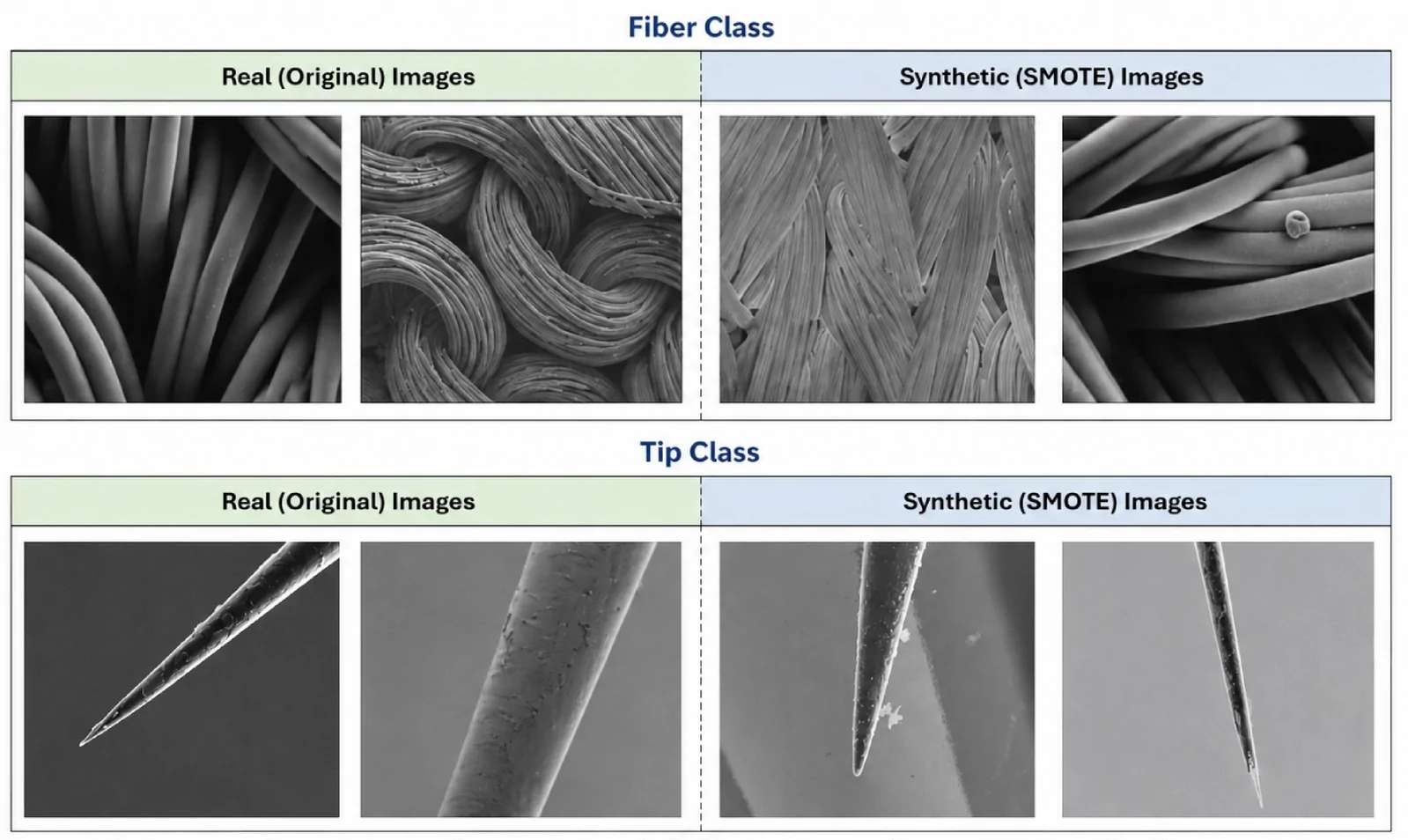
Representative examples of original SEM images and synthetic images generated using pixel-level SMOTE for the minority classes (Fiber and Tip).

To provide a qualitative assessment of the generated samples, representative examples of original and synthetic SEM images are presented in Fig. 4. Visual inspection indicates that the synthetic samples retain the overall morphological characteristics of the corresponding classes, including the elongated structures observed in fibers and the characteristic geometries of tip-like nanostructures. These synthetic images were generated exclusively for training data augmentation and were not included in the validation or test sets.

**Fig. 5:**
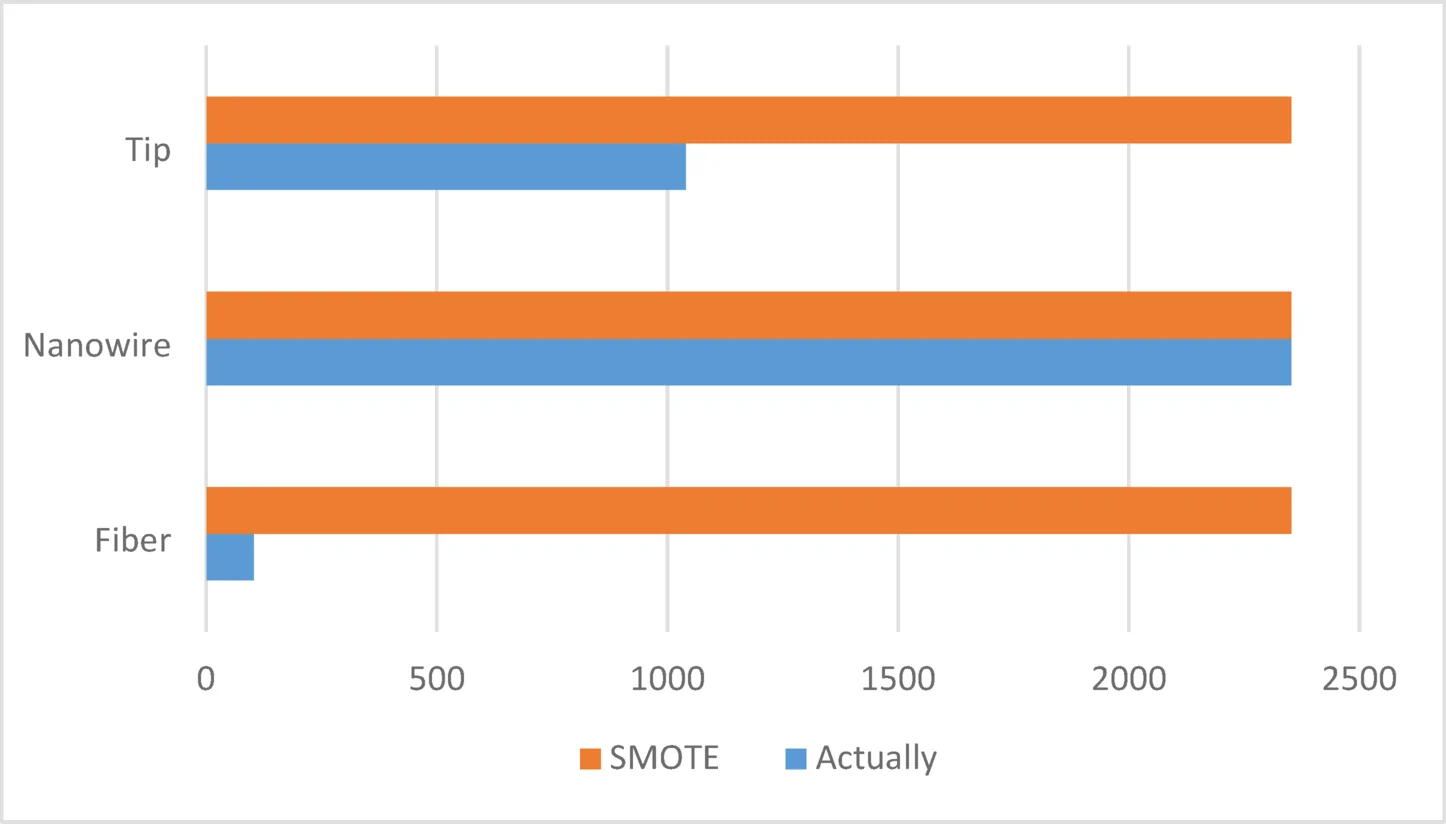
The classes’ distribution of the dataset before and after the SMOTE.

After applying the SMOTE technique exclusively to the training dataset, the number of instances in the Fiber, Nanowire, and Tip classes is 2352, 2352, and 2352 respectively. Figure 5 provides a comparison between the number of image samples before and after applying the SMOTE technique.

#### Feature-level SMOTE

In addition to the pixel-based balancing strategy, a feature-level approach was implemented to investigate the effect of operating SMOTE in a learned representation space. In this approach, deep feature embeddings were first extracted from the preprocessed SEM images using a pre-trained ResNet50 model. The extracted features from the training set were then used as input to the SMOTE algorithm, which generated synthetic samples for the minority classes directly in the feature space. This allows interpolation between semantically meaningful representations rather than raw pixel intensities, potentially preserving structural patterns more effectively.

### Model training

In this study, a multi-class classification approach based on deep learning models was adopted to classify SEM images of NFT objects. To investigate the impact of data representation on model performance, two training model performance were adopted based on the way SMOTE was applied: pixel-level representation and feature-level representation.

Three pre-trained CNNs: GoogLeNet, ShuffleNet, and SqueezeNet were utilized using a transfer learning strategy to leverage their powerful feature representation capabilities and adapt them to the NFT classification task. Each model was trained on the balanced SMOTE dataset to ensure performance improvements across all classes. This combination enables a comprehensive evaluation of performance versus efficiency trade-offs for multiclass SEM image classification.

*Transfer learning* Transfer learning (TL) is a technique in machine learning where a model trained for a certain task is used for another similar task. The advantage of this approach is that it significantly reduces training time and computational effort, while also providing better generalization in a low-data regime. Fine-tuning and feature extraction are two popular methods of transfer learning.

In fine-tuning, the pre-trained model weights are used as initial values and are updated during training on the target dataset. This process allows the model to adapt both low-level and high-level features from generic representations learned on large-scale datasets (e.g., ImageNet) to domain-specific features relevant to SEM images. The objective of fine-tuning is to refine the learned feature representations to better capture the morphological characteristics of the target classes, rather than relying solely on fixed generic features.

Feature extraction involves using a pre-trained model on a standard dataset, like ImageNet, but removing the top layer, which is used for classification. It then trains a new classifier to conduct classification on top of the pre-trained model. Yosinski et al. (2014) demonstrated that pre-trained models can effectively transfer learned representations to new tasks, highlighting the importance of transfer learning in deep learning applications^34^.

A feature is often considered the fundamental unit in computer vision and image processing, indicating whether a specific region of an image contains certain characteristics. These features can include specific structures such as pixels, edges, motifs, elements, or objects.

To evaluate models trained using fine-tuning and feature extraction, *categorical cross-entropy* is employed as the loss function. This function measures the discrepancy between the predicted and true class probabilities:

1 $$L\left( {y,\hat{y}} \right) = - \frac{1}{N} \mathop \sum \limits_{i = 1}^{N} \mathop \sum \limits_{j = 1}^{C} y_{i,j} \cdot log \left( {\hat{y}_{i,j} } \right)$$

here *N* is the batch size, *C* is the number of classes, $y_{i,j}$ = *1* if the correct label for sample* i* is *j* and zero otherwise, $y_{i,j}$ is the predicted probability of the *j* class for the *i*-sample passed through *softmax*.

The formula for the *softmax* function *z* is the vector of length *C* (number of classes):

2 $$Softmax\left( z \right)_{i} = \frac{{e^{{z_{i} }} }}{{\mathop \sum \nolimits_{k = 1}^{c} e^{{z_{k} }} }}$$

where $Softmax\left( z \right)_{i}$ denotes the predicted probability of class *i*, *e* is Euler’s number, and $\left( {\boldsymbol{z}} \right)_{{\boldsymbol{i}}}$ is the logit corresponding to class *i,* and *k* is the summation index over all classes. The denominator normalizes the exponentiated logits so that the output probabilities sum to one.

#### Training with pixel-level SMOTE

During the training phase, transfer learning was applied using pre-trained models (ShuffleNet, SqueezeNet, and GoogLeNet) initialized with ImageNet weights. Full fine-tuning was adopted, whereby all network layers were unfrozen and updated from the first training epoch. No layer freezing or progressive unfreezing strategy was employed. This approach allowed the pre-trained feature representations to adapt more effectively to the characteristics of SEM images. The final classification layer of each model was replaced with a fully connected layer consisting of three output neurons corresponding to the target NFT classes.

**Fig. 6:**
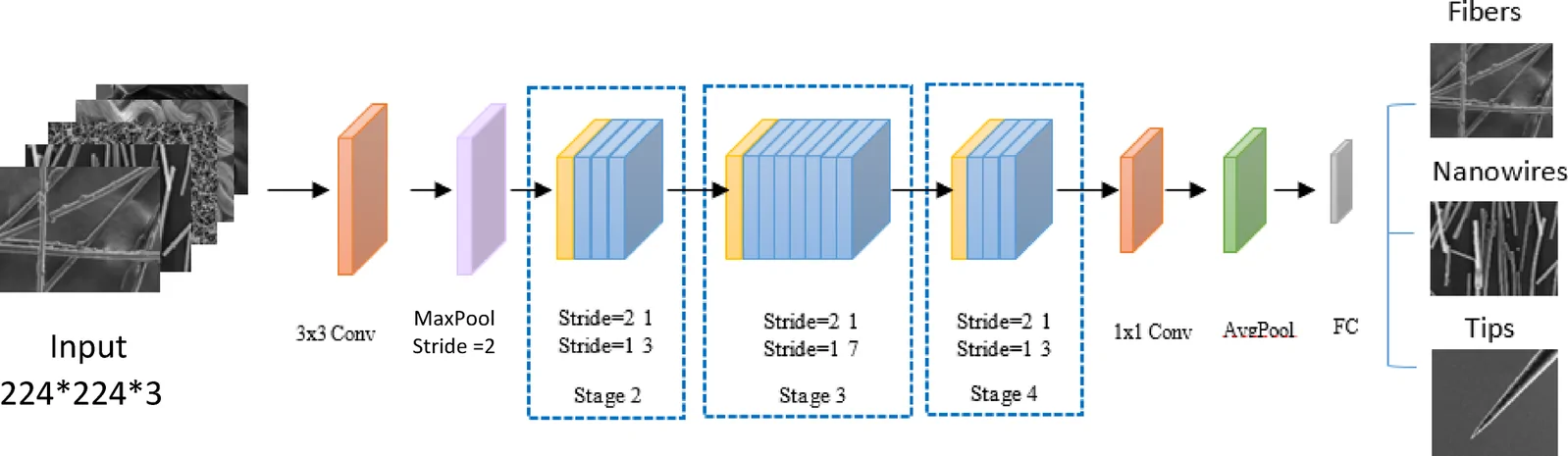
Architecture of The ShuffleNet Model.

*ShuffleNet* ShuffleNet is a CNN architecture designed for computation economy, especially under resource constraints—mobile accelerators/edge computing platforms. It was initially proposed by Zhang et al. (2018) to trade off the speed and accuracy of the deep learning models by reducing the parameter count and computational load^35^. A pre-trained ShuffleNetV2 model with a width multiplier factor of 1.0 is used in this work. The model is initialized with ImageNet Weights to benefit from previously learned visual features. To customize the model for three-class classification, a new fully connected layer of the model is replaced with a linear layer and constructs three numbers representing class probabilities. This adaptation makes the model compatible with the classification task while making it more practical. Figure 6 shows the framework of ShuffleNetV2.

**Fig. 7:**
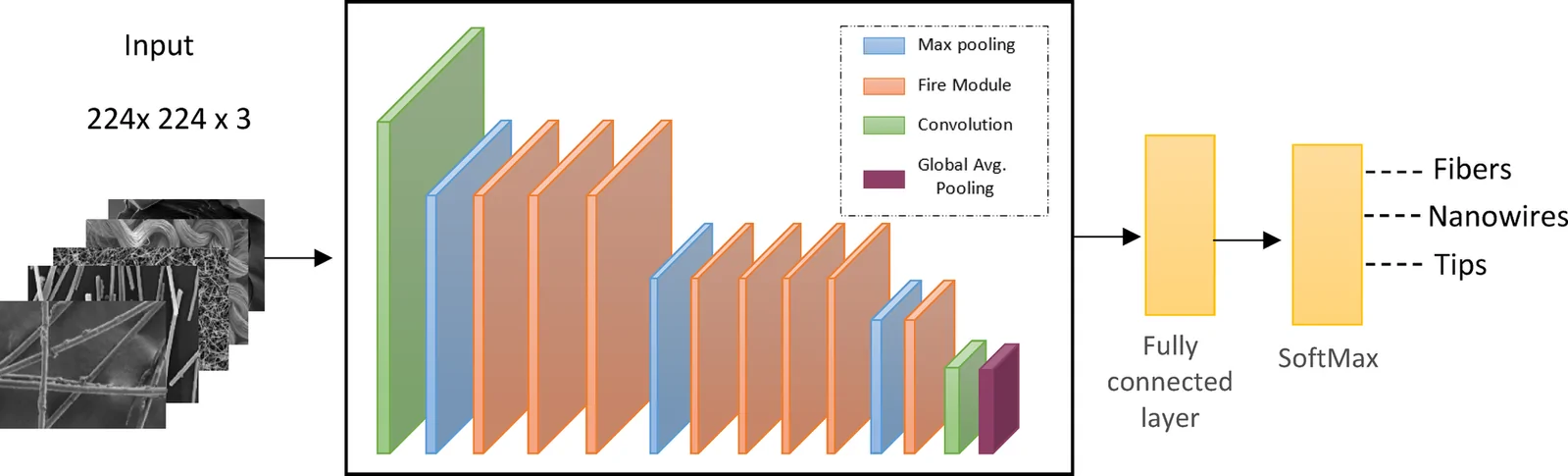
Architecture of The SqueezeNet Model.

*SqueezeNet* SqueezeNet is a lightweight CNN architecture designed for image classification with a low number of parameters. It is proposed to address the increasing demand for lightweight models to be deployed on mobiles, IoTs^36^. In our experiments, a pre-trained SqueezeNet 1.0 model with a width multiplier of 1.0 is adopted. For each model, all layers were fine-tuned during training to allow the model to adapt to the SEM image domain. This approach ensures the pre-trained knowledge is kept, as well as further adjusts towards the node three-class classification problem. The last layer, located in the classifier part of SqueezeNet, is substituted with a new 1 × 1 convolution layer with three output channels, equal to the number of target classes. This adaptation makes the model design consistent with the task specification. The new layer is randomly initialized. Figure 7 presents the architecture of the modified SqueezeNet model.

**Fig. 8:**
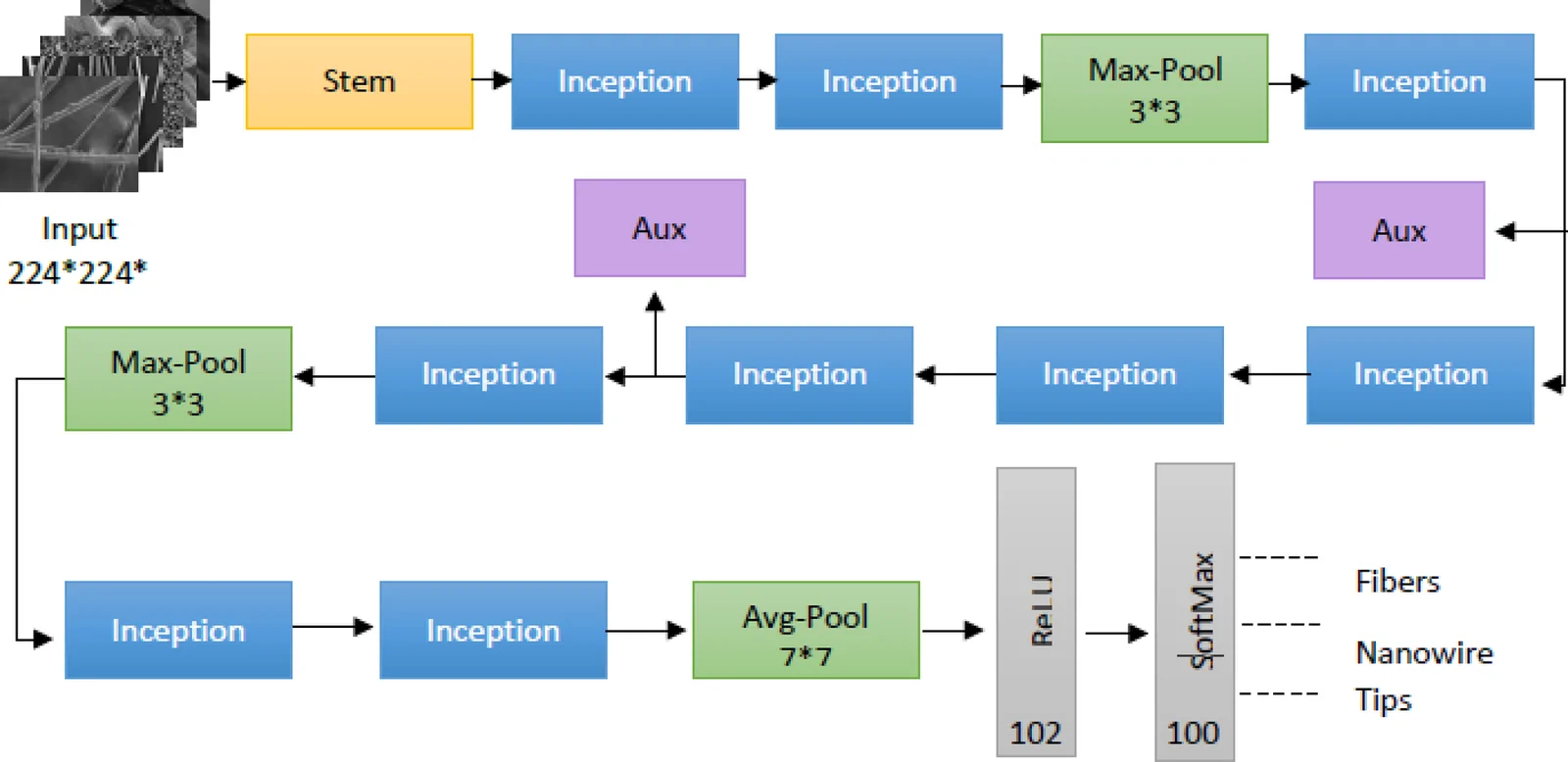
Architecture of The GoogLeNet Model.

*GoogLeNet* GoogLeNet (Inception v1) is a deep CNN, developed by Google’s research team for the ImageNet Large Scale Visual Recognition Challenge (ILSVRC) 2014 (Szegedy et al., 2015). The key novelty of GoogLeNet is the use of the inception modules, which support variable-size filters that allow the network to capture information at different spatial resolutions. The method achieves efficient computation and feature representation by combining receptive fields with various scales into the same layer^37^. A pre-trained GoogLeNet model is loaded, and the last fully connected layer is replaced by a new linear layer for three output classes. The new layer is set to have the same number of input features as the existing network structure. This adaptation gets GoogLeNet ready for the three-class classification moves, and it serves well for both fine-tuning and training from scratch. The new architecture enhances the generalization of the model and also the prediction accuracy of the model on datasets with well-separated classes. The architecture of the reformed GoogLeNet model is shown in Fig. 8. Table 3 shows the main differences between the three models used in the proposed approach.

**Table 3:** Summary of the main differences between the three models used.

Model	Architecture highlights	Advantages	Parameters (Approx.) (M)
ShuffleNet	Uses pointwise group convolution and channel shuffling for efficiency	High speed, low computational cost, good accuracy	~ 1.0
SqueezeNet	Employs “fire modules” combining 1 × 1 and 3 × 3 filters	Extremely small size with competitive performance	~ 1.2
GoogLeNet	Deep CNN with inception modules for multi-scale feature extraction	High accuracy, strong feature representation	~ 6.8

Prior to training, class imbalance in the training set was addressed using the Synthetic Minority Over-sampling Technique (SMOTE). The algorithm was implemented using k = 5 nearest neighbors, corresponding to the default configuration of the imbalanced-learn library. Synthetic samples were generated only for the minority classes within the training set, while the validation and test sets remained unchanged to avoid data leakage.

Model training was conducted using the categorical cross-entropy loss function (Eq. 1) which evaluates the difference between predicted probabilities and ground truth labels, a softmax activation function (Eq. 2) was applied to the output layer to generate class probabilities.

For optimization, stochastic gradient descent (SGD) was used with a learning rate of 0.01 and a batch size of 64. The models were trained for 5, 10, 15, and 20 epochs to examine performance across different training durations.

To prevent overfitting, an early stopping strategy was based on validation accuracy was implemented. After each epoch, the model was evaluated on the validation set. Whenever an improvement in validation accuracy was observed, the model weights were saved as the best-performing checkpoint and the patience counter was reset. Otherwise, the patience counter increased. Training was stopped when no improvement was observed for a predefined number of consecutive epochs. The model used for final evaluation corresponds to the checkpoint with the highest validation accuracy.

#### Training with feature-level SMOTE

SMOTE was applied after feature extraction instead of raw pixel data. A pre-trained ResNet50 model was employed as a fixed feature extractor by removing its final classification layer. Each input image was passed through the network to obtain a high-level feature vector representing its semantic content. These extracted feature vectors were then used as input to the SMOTE algorithm to generate synthetic samples for the minority classes. The SMOTE algorithm was implemented using k = 5 nearest neighbors, corresponding to the default configuration of the imbalanced-learn library. Unlike the pixel-based approach, the generated data remained in feature space and were not transformed back into image format.

ResNet-50 is a deep convolution neural network that introduces residual learning through shortcut (skip) connections, allowing the network to effectively train very deep architectures without suffering from vanishing gradient problems. Instead of learning direct mapping, the network learns residual functions, which improves convergence stability and enhances feature representation quality.

**Fig. 9:**
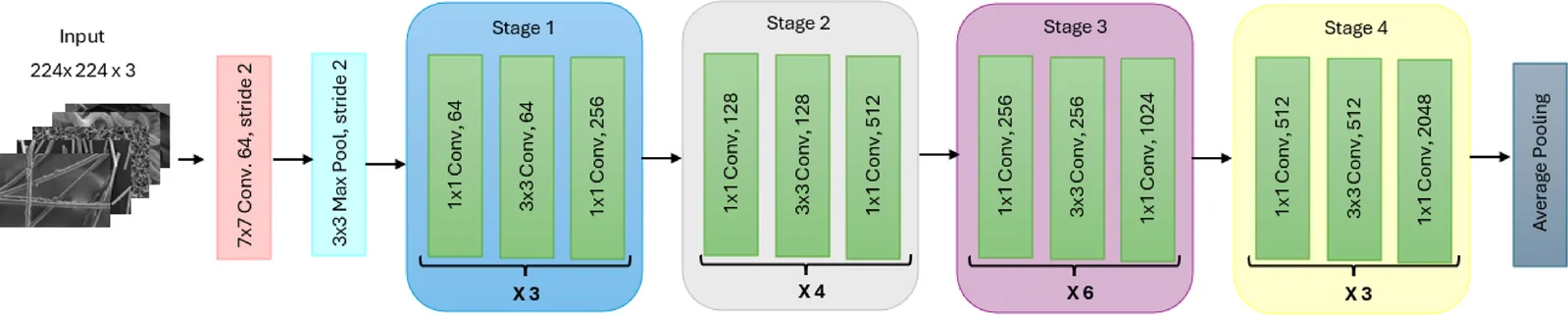
Architecture of The ResNet50 Model.

Due to its strong capability in capturing hierarchical visual features—from low-level edges to high-level semantic patterns—ResNet50 has been widely adopted as a feature extractor in transfer learning tasks. In this work, the model was initialized with ImageNet pre-trained weights, and its convolutional layers were used to extract discriminative features from SEM images. The output of the final pooling layer was flattened into a feature vector, which served as the input for the SMOTE algorithm. As shown in Fig. 9.

In this study, ResNet50 was used as a fixed feature extractor and its weights were kept frozen during training. This design was adopted to isolate the impact of feature-level SMOTE and evaluate the effectiveness of balancing the extracted feature representations independently from end-to-end network adaptation. Future work will investigate the integration of feature-level SMOTE with full ResNet50 fine-tuning.

The extracted feature vectors obtained from ResNet50 (2048-dimensional features) were used as input to a Multi-Layer Perceptron (MLP) classifier. The MLP architecture consisted of two hidden fully connected layers containing 512 and 256 neurons, respectively. ReLU activation functions were applied after each hidden layer. Batch normalization was employed after the first hidden layer to improve training stability, while dropout regularization with rates of 0.4 and 0.3 was applied to the first and second hidden layers, respectively, to reduce overfitting. The output layer consisted of three neurons corresponding to the NFT classes. The model was trained using the Adam optimizer with a learning rate of 1 × 10⁻^4^, a batch size of 32, and a categorical cross-entropy loss function for 50 epochs.

## The experimental results analysis

This section presents the results obtained from a series of experiments conducted to evaluate the performance of multiple neural network architectures for the detection of NFT objects in scanning electron microscopy (SEM) images. To address the issue of class imbalance, the Synthetic Minority Over-sampling Technique (SMOTE) was employed, ensuring a more balanced training dataset and improving classification robustness.

The proposed method was implemented and trained on Google Colab, utilizing its freely available GPU resources. Each experimental run was allocated 12 GB of RAM and executed on an NVIDIA Tesla K80 GPU, with a maximum continuous runtime of up to 12 h. This computational environment provided sufficient capacity for model training, parameter tuning, and comparative evaluation across different architectures.

### Performance metrics

To measure the performance of classification models, the following metrics are used:

3 $$Accuracy = \frac{TP + TN}{{TP + TN + FP + FN}}$$

4 $$Recall = \frac{TP}{{TP + FN}}$$

5 $$Precission = \frac{TP}{{TP + FP}}$$

6 $$F1{\mathrm{-}}Score = \frac{{2\left( {Recall*Precission} \right)}}{Recall + Precission}$$

where *TP*, *TN*, *FP*, and *FN* stand for True Positive, True Negative, False Positive, and False Negative, respectively. *TP* is the model correctly predicted instances of a class; *TN* are instances that were correctly predicted as not belonging to the given class; *FP* are instances that were incorrectly predicted as the given class; and *FN* are instances that belong to the given class but were incorrectly predicted as another class. After the models finished training, they were tested on a test set to evaluate the model’s accuracy. The models were tested on 33, 735, and 325 images in the Fiber, Nanowire and Tip classes, respectively. Overall classification accuracy (Eq. 3), recall (Eq. 4) (sometimes called sensitivity), precision (Eq. 5), and F1-score (Eq. 6) were used to evaluate the model performance. A confusion matrix was plotted to illustrate the distribution of correctly and incorrectly classified images, and a classification report was obtained using the scikit-learn metrics library.

### Testing accuracy and confusion matrix

The performance of the models SqueezeNet, ShuffleNet, and GoogLeNet was evaluated on both imbalanced datasets and datasets balanced using SMOTE. The analysis was carried out across different epochs to determine the impact of training duration on accuracy, precision, recall, F1-score, and AUC.

#### Results on imbalanced dataset

On the imbalanced dataset, GoogLeNet achieved the highest performance, reaching an accuracy of 98.17% and F1-score of 95.01 at 15 epochs. ShuffleNet demonstrated competitive performance with an accuracy of 98.08% and a slightly higher F1-score of 95.3% at the same epoch. SqueezeNet showed comparatively lower performance, achieving an accuracy of 97.26% and an F1-score of 94.61% at 10 epochs. Table 4 presents a comparison of the three models (SqueezeNet, ShuffleNet, and GoogLeNet) by showing their accuracy, precision, recall, F1-score, AUC, and training time across different epochs on the imbalanced dataset.

**Table 4:** Performance of SqueezeNet, ShuffleNet, and GoogLeNet on the imbalanced dataset.

Models	No. of epochs	Accuracy (%)	Time	Precision (%)	Recall (%)	F1-score (%)	AUC (%)
SqueezeNet	5	96.07	5 min 35 s	92.16	93.39	92.76	96.14
	10	97.26	5 min 36 s	94.55	94.72	94.61	98.38
	15	97.16	6 min 37 s	95.72	93.08	94.34	97.56
	20	96.98	8 min 3 s	93.13	95.04	94.05	97.95
ShuffleNet	5	95.61	4 min 47 s	63.72	65.59	64.64	99.54
	10	97.8	4 min 41 s	93.12	94.54	93.8	99.59
	15	98.08	4 min 24 s	94.7	95.92	95.3	99.8
	20	97.8	4 min 27 s	95.45	95.56	95.5	99.8
GoogLeNet	5	96.98	20 min 18 s	90.77	95.83	93.04	99.63
	10	96.16	11 min 28	87.09	94.8	90.39	99.36
	15	**98.17**	18 min 58 s	94.16	95.91	95.01	99.87
	20	96.89	17 min 44 s	89.17	92.15	90.58	99.48

Significant values are in [bold].

**Fig. 10:**
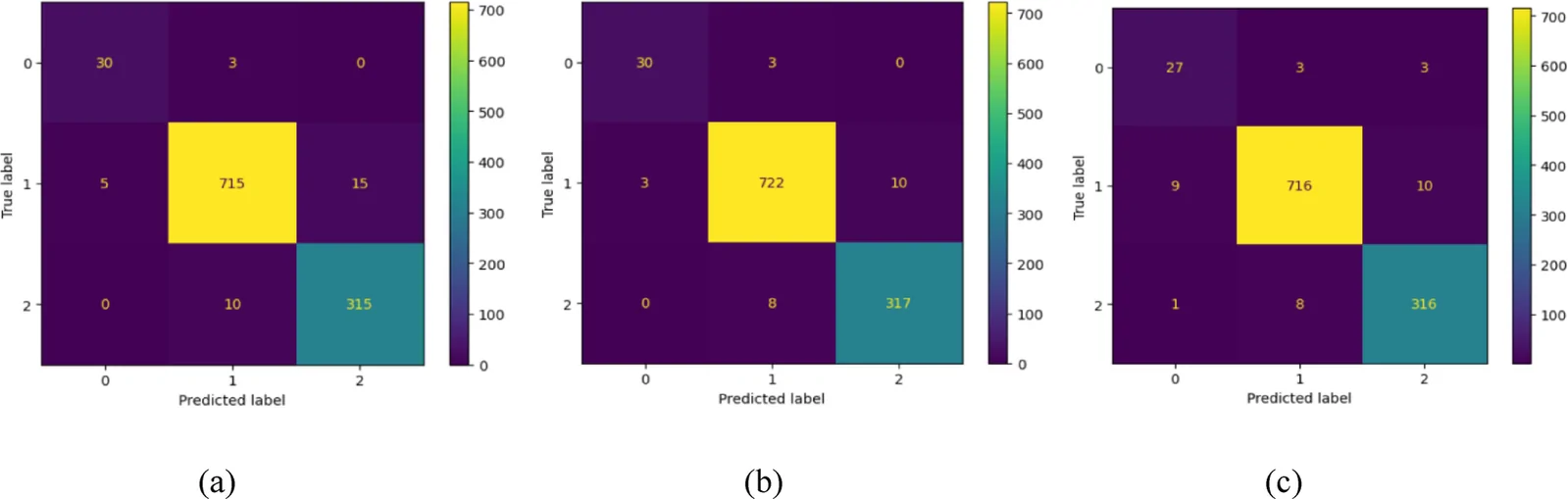
Confusion Matrix for (**a**) SqueezeNet, (**b**) ShuffleNet and (**c**) GoogLeNet on imbalanced dataset.

The confusion matrix helps to understand in more detail how the model’s predictions are made in the imbalanced dataset. It presents the distribution of true and False-Positive predictions for the classes Fiber, Nanowire, and Tip. The confusion matrices reveal that all models were biased toward the majority class (Nanowire), resulting in higher misclassification rates for minority classes (Fiber and Tip). This highlights the challenges associated with class imbalance. The confusion matrices of GoogLeNet, ShuffleNet, and SqueezeNet on the imbalanced dataset at 20 epochs are shown in Fig. 10.

**Fig. 11:**
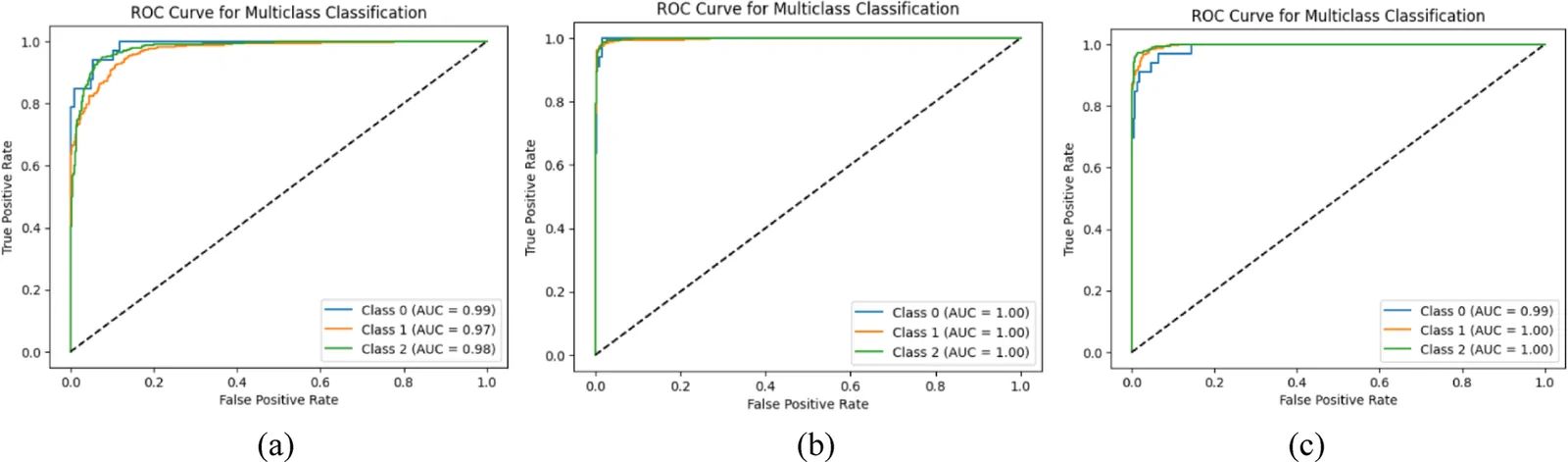
AUC curve for (**a**) SqueezeNet, (**b**) ShuffleNet and (**c**) GoogLeNet on imbalanced dataset.

The AUC curve quantifies the model’s capacity to distinguish three classes under the condition of the imbalanced dataset. The AUC-ROC score was computed using a one-vs-rest (OvR) strategy with weighted averaging to account for class imbalance. GoogLeNet and ShuffleNet reached an AUC of 99.63% and 99.54%, respectively, while SqueezeNet achieved 97.95%, after 20 epochs, which is like the other models (achieved theoretically high values in the same number of epochs), but overall performed less well. As shown in Fig. 11.

**Fig. 12:**
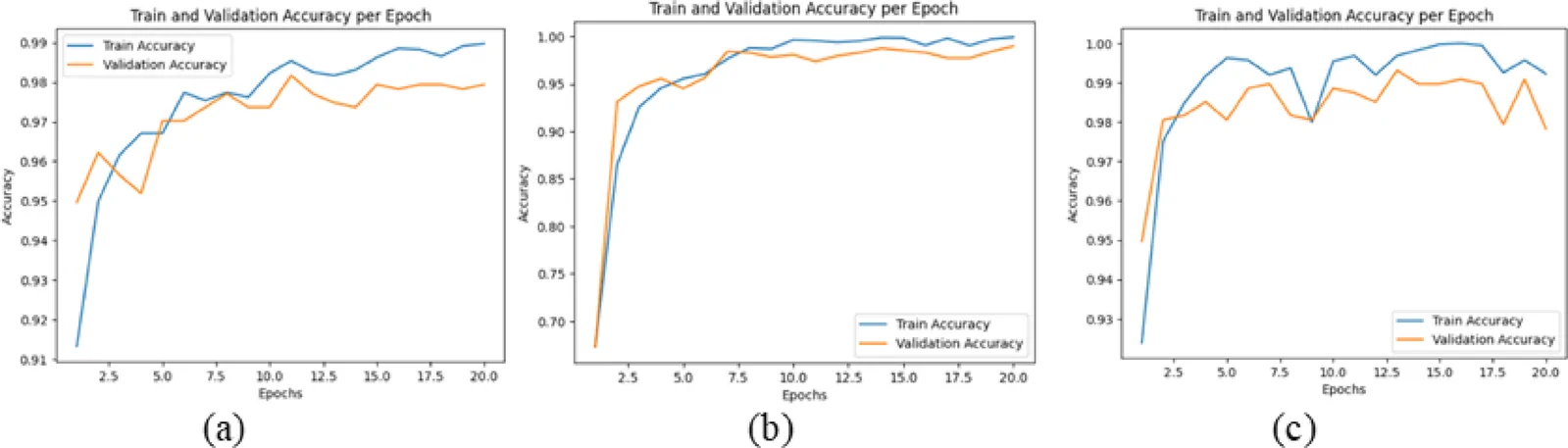
Training and validation accuracy plots (**a**) SqueezeNet, (**b**) ShuffleNet, and (**c**) GoogLeNet on the imbalanced dataset.

The training and validation accuracy plots further reveal the learning dynamics of the models. SqueezeNet converged quickly but exhibited a large gap between training and validation accuracy after 10 epochs, suggesting overfitting to the majority class due to dataset imbalance. Around 10 epochs, ShuffleNet converged faster than GoogLeNet and kept a smaller gap between training and validation accuracies, making it a relatively balanced performer. GoogLeNet required more time to converge but achieved the most consistent training and validation accuracy after 15 epochs, demonstrating stronger generalization at larger epochs. These trends are illustrated in Fig. 12.

**Fig. 13:**
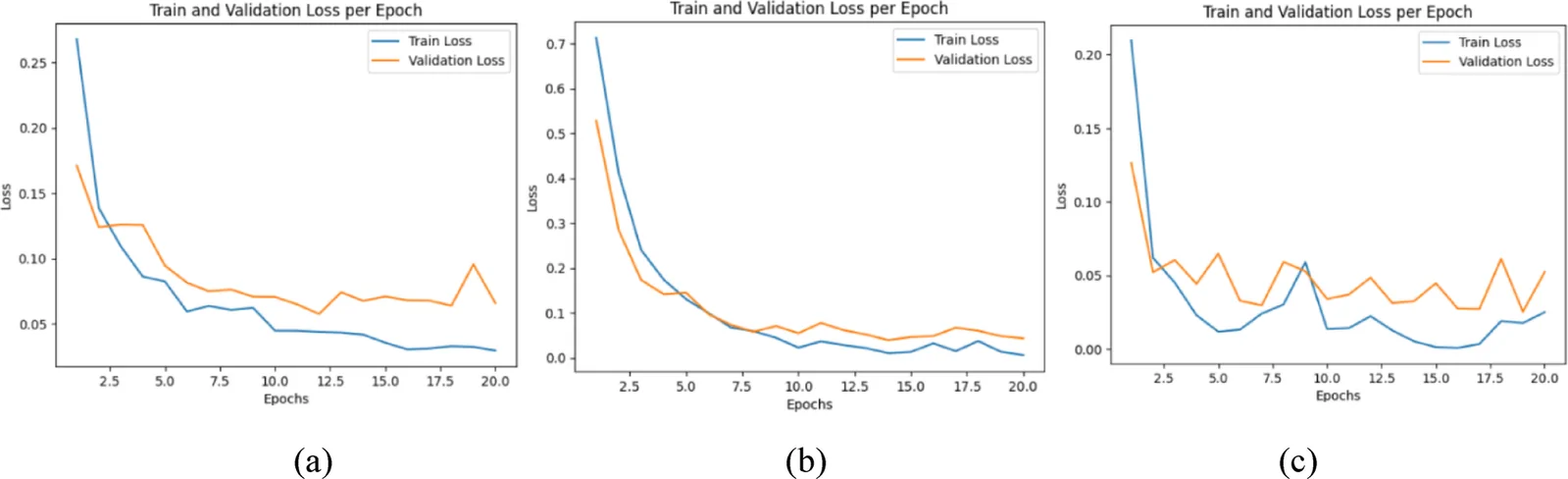
Training and validation loss plots (**a**) SqueezeNet, (**b**) ShuffleNet, and (**c**) GoogLeNet on the imbalanced dataset.

The training and validation loss curves provide insight into the learning behavior under class imbalance. As shown in Fig. 13, all models exhibit a decrease in training loss; however, a noticeable divergence between training and validation loss appears after several epochs. This gap is more pronounced in SqueezeNet, indicating overfitting toward the dominant class (Nanowire). ShuffleNet shows relatively stable behavior with a smaller gap, while GoogLeNet demonstrates a more gradual convergence but still reflects the negative impact of data imbalance on generalization performance.

#### Results after balancing with SMOTE

This section presents the classification results obtained after addressing class imbalance using SMOTE under two different data representations. The performance is evaluated separately for each approach to highlight the effect of data representation on model behavior. The results obtained using pixel-level SMOTE are first reported, where the models were trained directly on the balanced image dataset. This is followed by the results of feature-level SMOTE, where classification was performed using high-level feature representations extracted from a pre-trained network.

This organization allows for a clear and structured evaluation of both approaches prior to the comparative analysis presented in “Comparative analysis” section.

*Results using pixel-level SMOTE* SMOTE was applied to the training data to address class imbalance and improve model performance. After applying SMOTE, all models demonstrated significant improvements across all evaluation metrics. Among the tested architectures, GoogLeNet achieved the highest performance, reaching an accuracy of 98.35% and an F1-score of 98.34% at 20 epochs. ShuffleNet achieved comparable performance with an accuracy of 98.35% and an F1-score of 98.33%. SqueezeNet also improved, reaching an accuracy of 96.89% and an F1-score of 96.93% at 20. Table 5 summarizes the performance of SqueezeNet, ShuffleNet, and GoogLeNet on the balanced dataset. To evaluate potential overfitting, both training and validation metrics were monitored throughout the training process. The close alignment between validation and test results indicates good generalization capability despite the high AUC values. All performance metrics, including precision, recall, and f1-score, were computed using the weighted averaging scheme based on the same prediction outputs, and evaluated on a completely unseen test set that was not used during training or data balancing.

**Table 5:** Performance comparison of SqueezeNet, ShuffleNet, and GoogLeNet on the balanced dataset.

Models	No. of epochs	Accuracy (%)	Time	Precision (%)	Recall (%)	F1-score (%)	AUC (%)
SqueezeNet	5	95.7	13 min	95.9	95.7	95.75	99.43
	10	96.25	11 min 37 s	96.29	96.25	96.29	99.4
	15	95.97	12 min 22 s	96.13	95.97	96.02	99.47
	20	96.89	10 min 23 s	97	96.89	96.93	99.52
ShuffleNet	5	97.71	8 min 8 s	97.72	97.71	97.69	99.68
	10	97.71	8 min 15 s	97.70	97.71	97.70	99.72
	15	98.08	7 min 59 s	98.07	98.08	98.06	99.67
	20	**98.35**	8 min 18 s	98.34	98.35	98.33	99.69
GoogLeNet	5	97.26	39 min 22 s	97.22	97.26	97.23	99.79
	10	97.8	33 min 32 s	97.79	97.8	97.79	99.85
	15	98.08	33 min 23 s	98.09	98.08	98.06	99.87
	20	**98.35**	32 min 58 s	98.34	98.35	98.34	99.91

Significant values are in [bold].

**Fig. 14:**
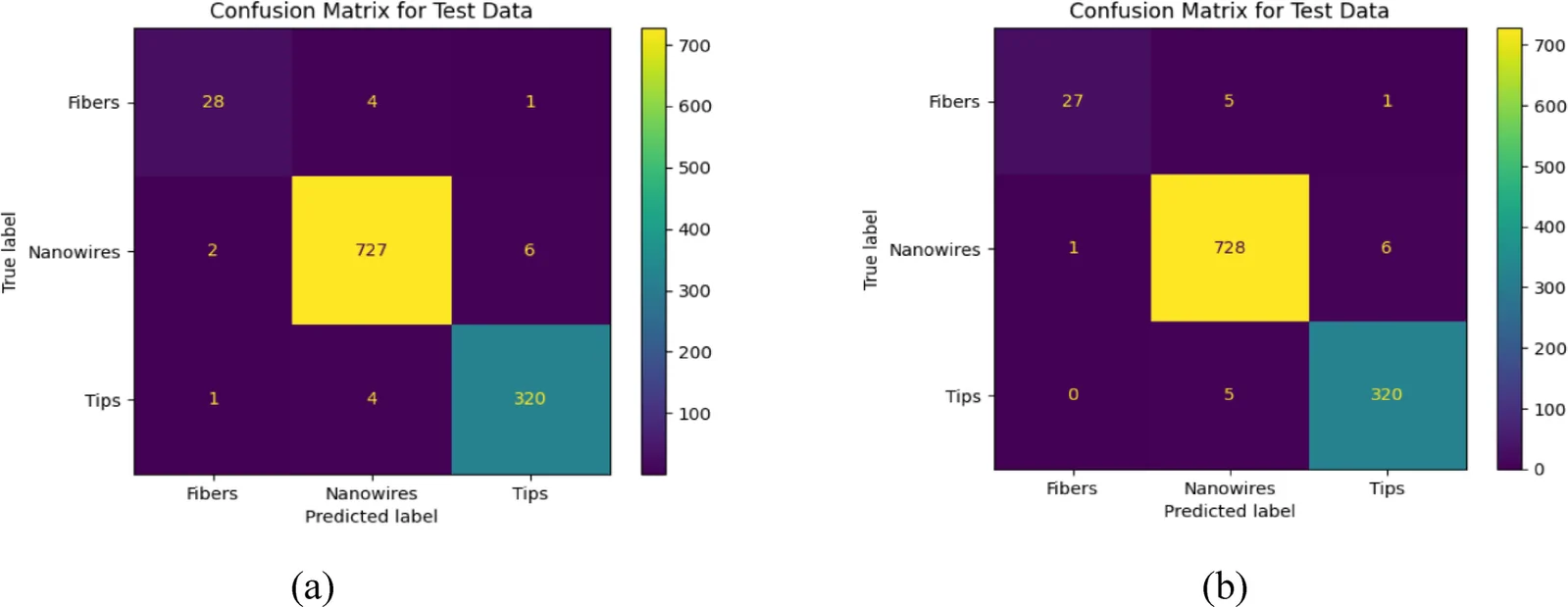
Confusion Matrix for (**a**) GoogLeNet, (**b**) ShuffleNet on the balanced dataset using SMOTE.

**Fig. 15:**
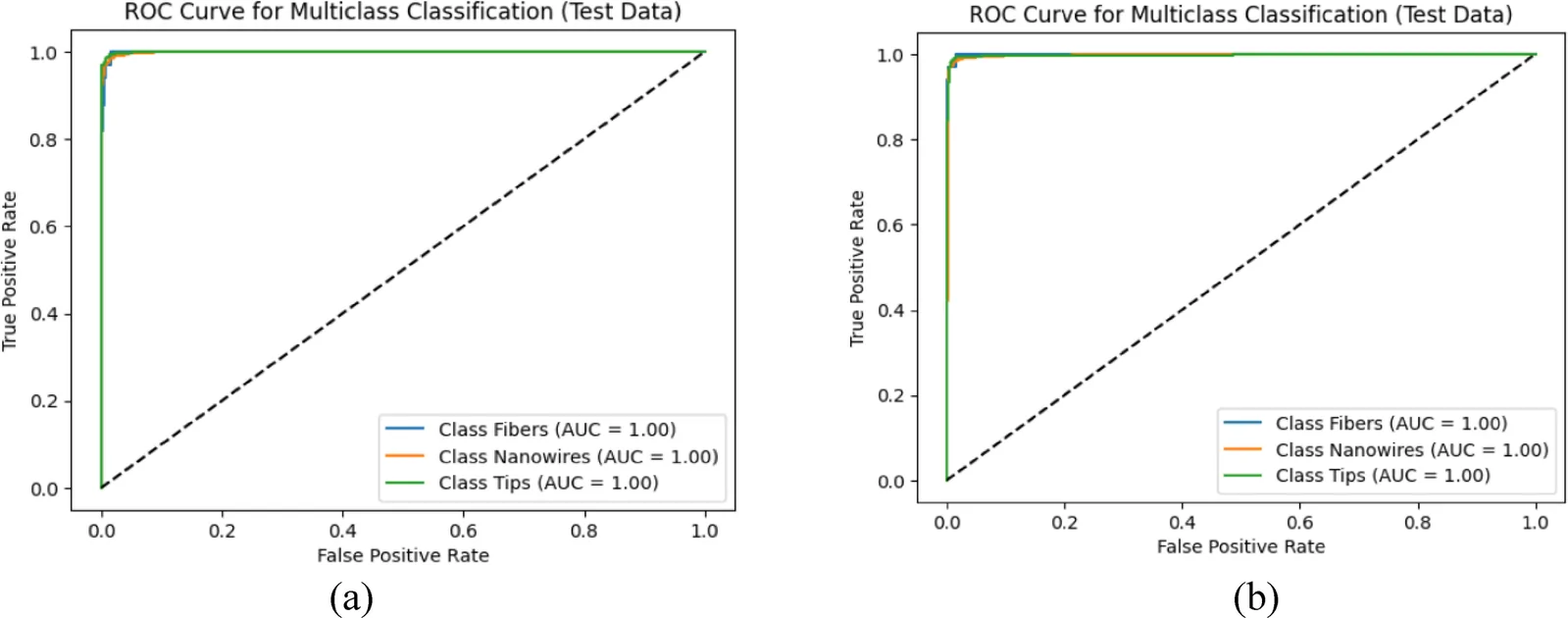
AUC curve for (**a**) GoogLeNet, (**b**) ShuffleNet on the balanced dataset using SMOTE.

**Fig. 16:**
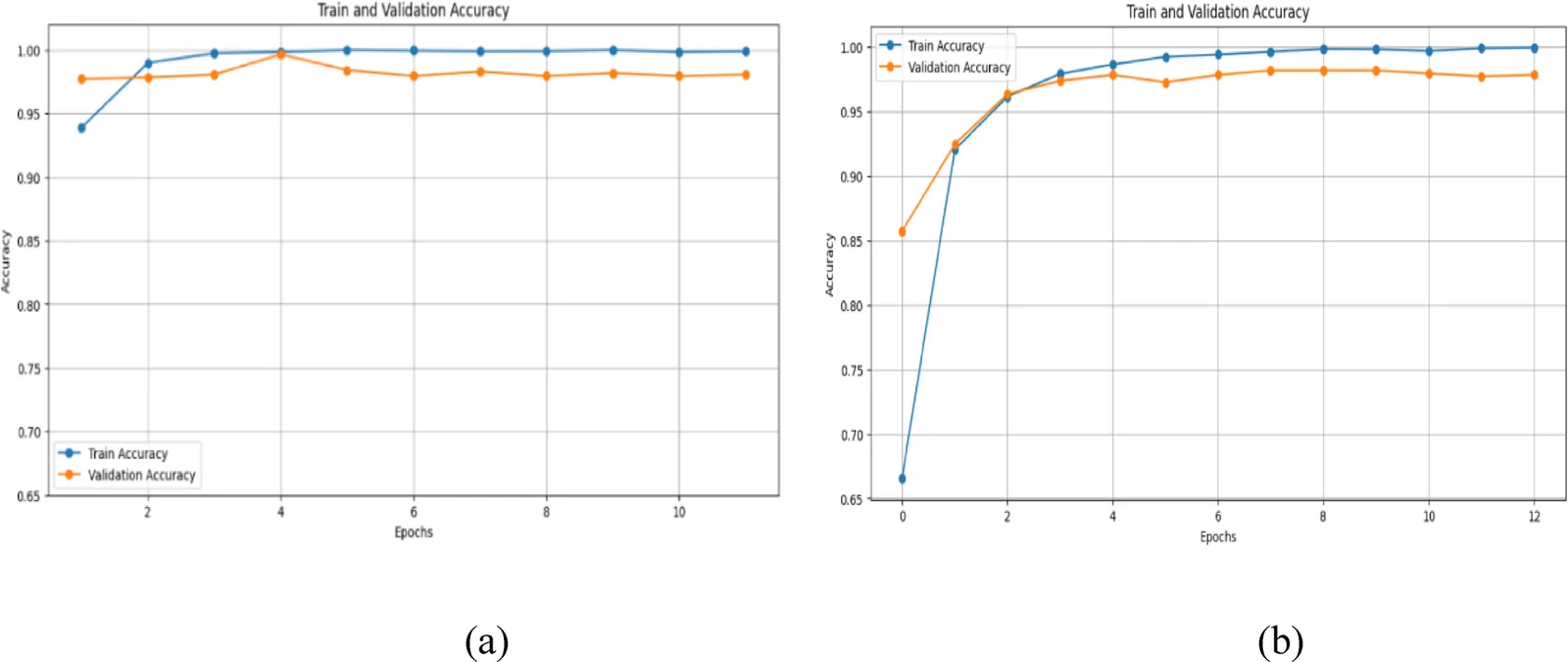
Training and validation accuracy plots (**a**) GoogLeNet, (**b**) ShuffleNet on the balanced dataset using SMOTE.

**Fig. 17:**
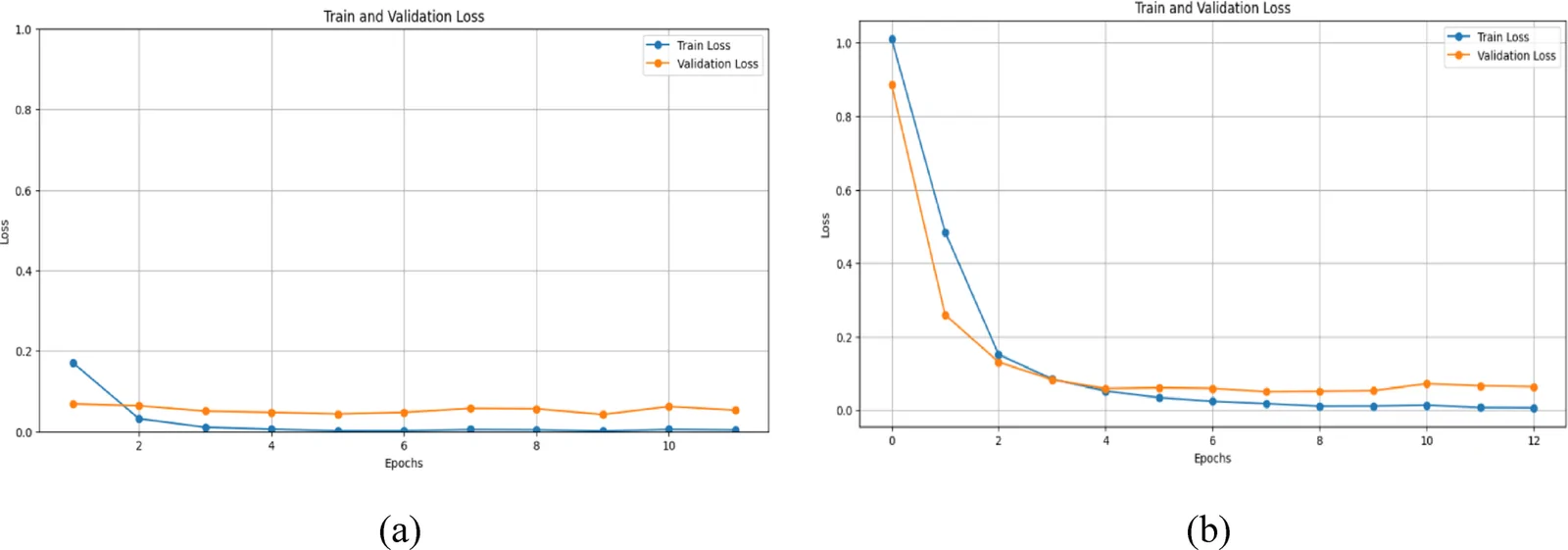
Training and validation loss plots (**a**) GoogLeNet, (**b**) ShuffleNet on the balanced dataset using SMOTE.

Figure 14 illustrates the confusion matrix for GoogLeNet and ShuffleNet on the balanced dataset after applying SMOTE at 20 epochs, highlighting significant improvements in the classification of minority classes. The AUC curve, depicted in Fig. 15, demonstrates the strong classification capability of the models, with GoogLeNet achieving the highest AUC of 99.91%. over 20 epochs, while ShuffleNet achieved a slightly lower percentage of 99.69% with SMOTE-applied data, emphasizing its robustness in multi-class classification tasks. Training and validation curves, as shown in Figs. 16 and 17, demonstrate improved convergence behavior and reduced overfitting after applying SMOTE. These results confirm the effectiveness of SMOTE in improving classification performance and model generalization. Table 5 provides a class-wise performance comparison of SqueezeNet, ShuffleNet, and GoogLeNet at 20 epochs, reporting accuracy, precision, recall, and F1-score for each class.

**Fig. 18:**
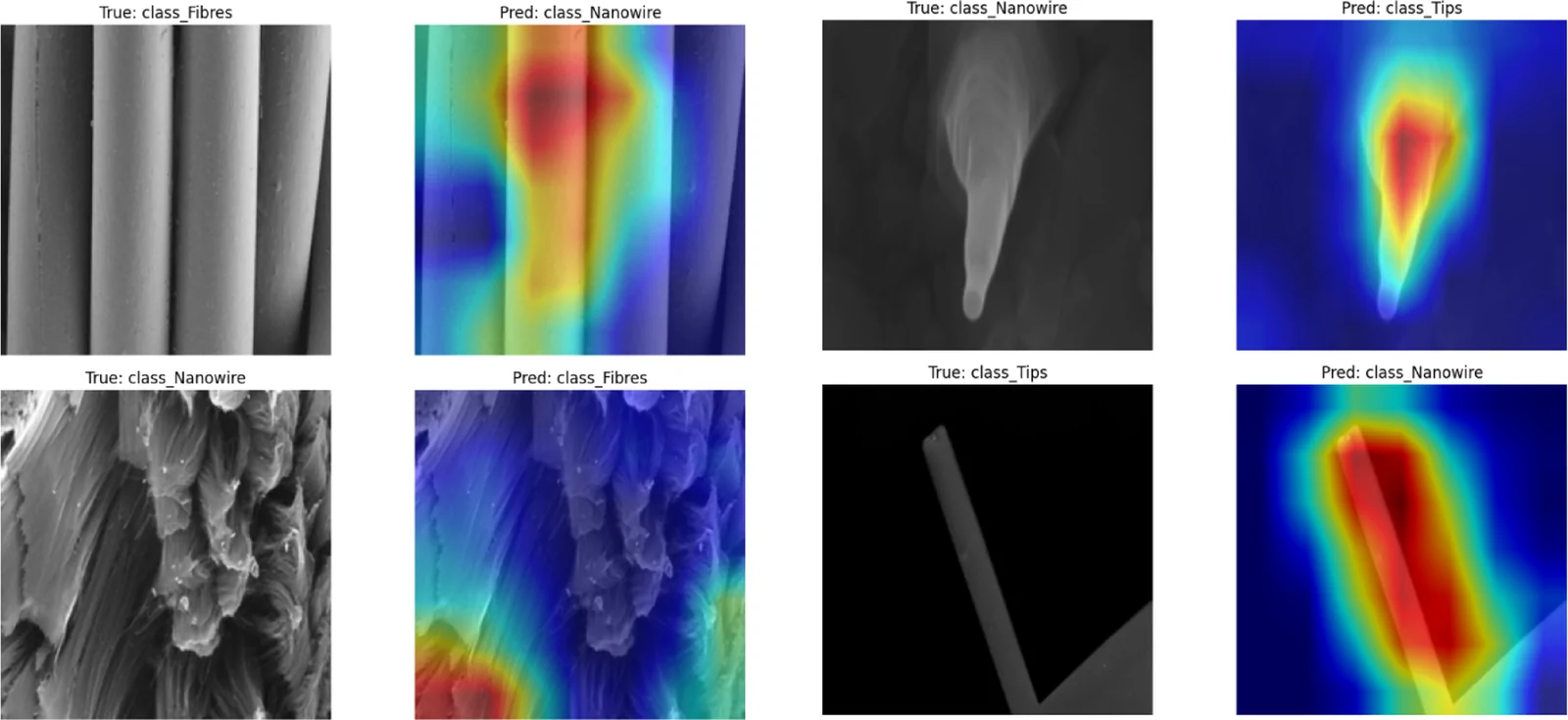
Presents examples of misclassified SEM images after applying SMOTE, highlighting the challenges in distinguishing between visually similar classes.

To further investigate the remaining classification errors after applying SMOTE, representative misclassified samples together with their corresponding Grad-CAM visualizations are presented in Fig. 18. The activation maps indicate that the models primarily focus on the dominant morphological structures of the nanomaterials when making predictions. The visual analysis reveals that most misclassifications occur between classes that share similar geometric characteristics. For example, elongated cylindrical structures may lead to confusion between Fibers and Nanowires, while pointed or tapered morphologies can result in confusion between Nanowires and Tips. This observation is consistent with the lower recall values obtained for the Fiber class Table 6, compared with the Nanowire and Tip classes.

**Table 6:** The performance of the models SqueezeNet, ShuffleNet, and GoogLeNet for each class at 20 epochs.

Models	Class	Accuracy (%)	Precision (%)	Recall (%)	F1-score (%)
SqueezeNet	Fibers	98.9	76.92	90.91	83.33
	Nanowires	96.98	98.08	97.41	97.75
	Tips	97.9	96.6	96.31	96.46
ShuffleNet	Fibers	99.36	96.43	81.82	88.52
	Nanowires	98.44	98.64	99.05	98.85
	Tips	98.9	97.86	98.46	98.16
GoogLeNet	Fibers	99.27	90.32	84.85	87.5
	Nanowires	98.54	98.91	98.91	98.91
	Tips	98.9	97.86	98.46	98.16

Overall, the Grad-CAM analysis confirms that the models rely on meaningful structural features rather than irrelevant image regions. The remaining errors are therefore mainly attributable to the intrinsic morphological similarity among the NFT classes rather than to class imbalance, indicating that SMOTE substantially mitigates the imbalance problem while the residual challenges arise from the visual complexity of SEM image classification.

*Results using feature-level SMOTE* In this approach, high-level features were first extracted using a pre-trained ResNet50 model, where the final classification layer was removed to obtain compact feature vectors for each image. SMOTE was then applied to these extracted feature vectors to generate synthetic samples for the minority classes. The balanced feature set was subsequently used to train a Multi-Layer Perceptron (MLP) classifier.

The training process demonstrated stable convergence, with validation accuracy steadily improving across epochs. The model achieved a final accuracy of 97.44%, with an F1-score of 97.45%, precision of 97.52%, recall of 97.44%, and an AUC score of 99.76%. These results indicate that feature-level SMOTE can effectively handle class imbalance while leveraging high-level semantic representations extracted from deep neural networks.

**Fig. 19:**
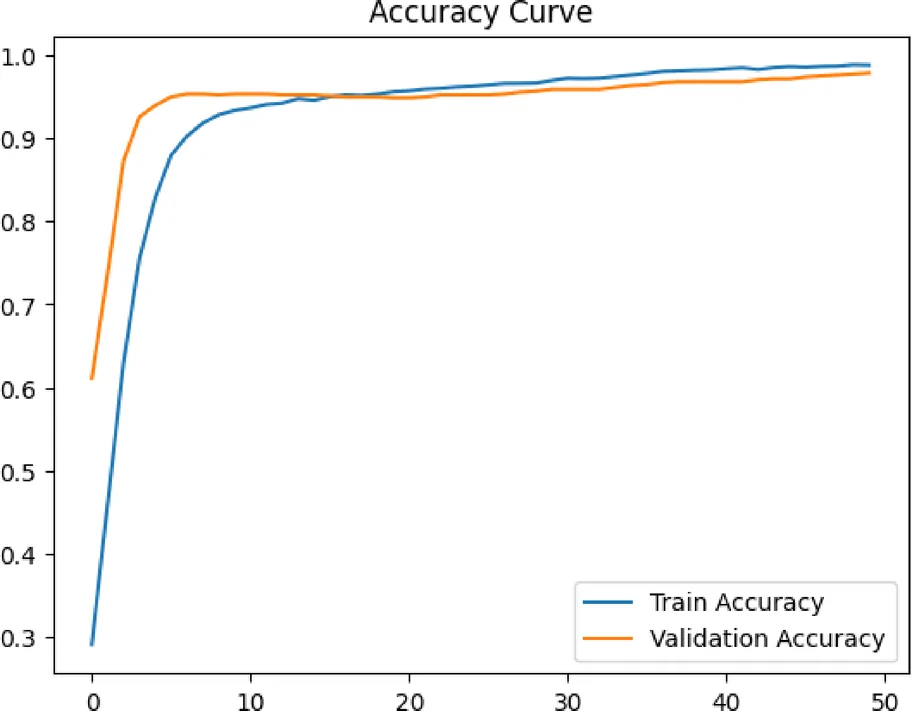
Training and validation accuracy plots using feature-level SMOTE.

**Fig. 20:**
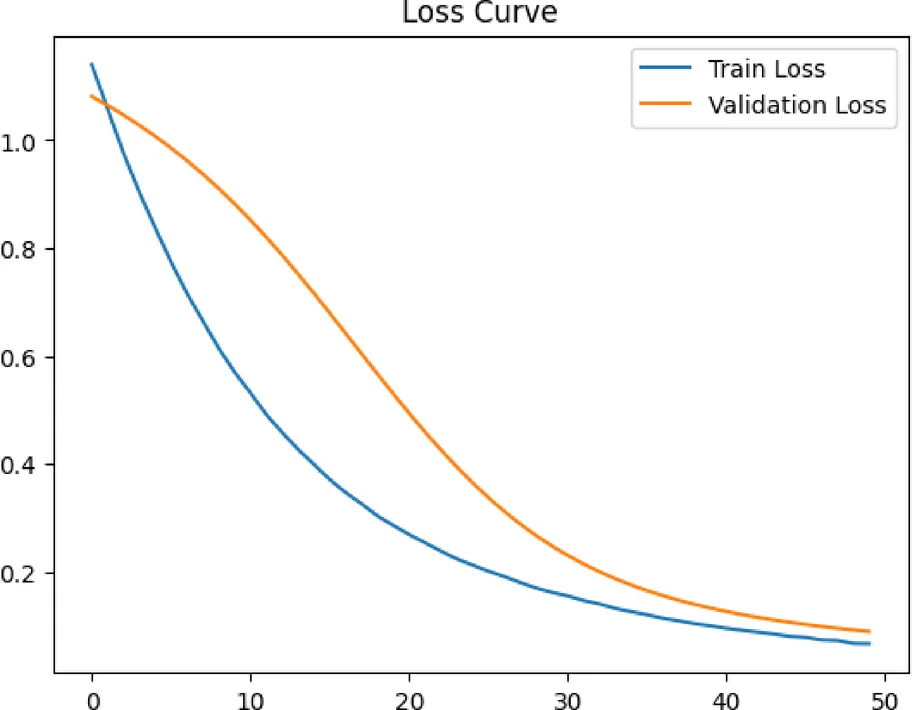
Training and validation loss plots using feature-level SMOTE.

The training and validation accuracy curves demonstrate a steady improvement in model performance across epochs, with validation accuracy increasing consistently and closely following the training accuracy, as shown in Fig. 19. This behavior indicates effective learning and good generalization capability without significant overfitting. Similarly, the training and validation loss curves depicted in Fig. 20 show a gradual decrease, confirming stable convergence during training.

**Fig. 21:**
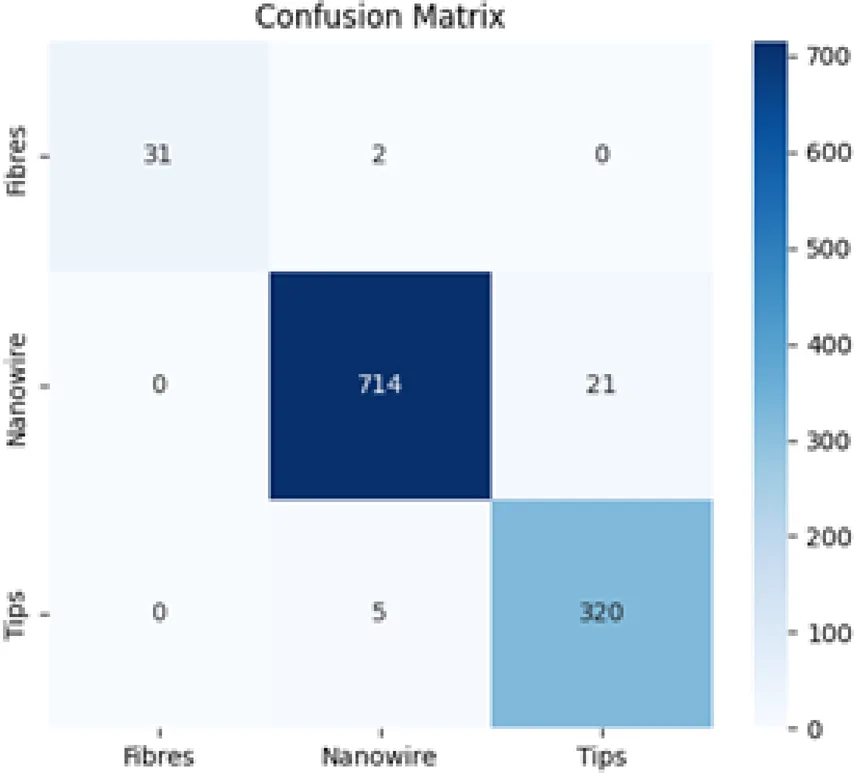
Confusion matrix for feature-level SMOTE classification results.

**Fig. 22:**
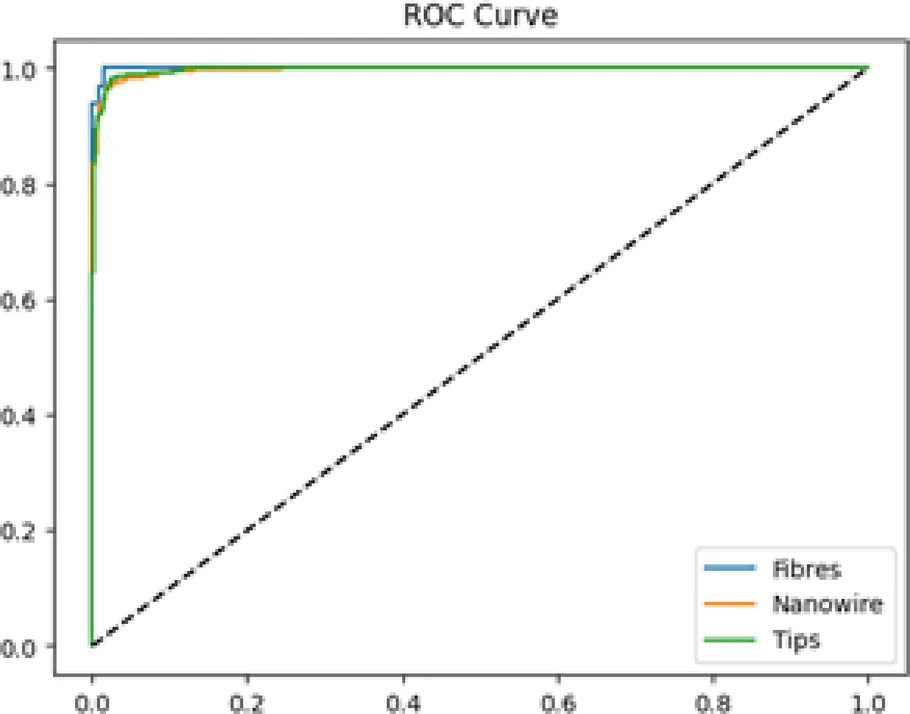
ROC curves for feature-level SMOTE across all classes.

Figure 21 presents the confusion matrix, which provides a detailed view of class-wise predictions. Most samples across the three classes (Fiber, Nanowire, and Tip) were correctly classified, while the limited misclassifications mainly occurred between structurally similar classes, which is expected given the visual similarity of SEM images. The ROC curves further demonstrate the strong discriminative capability of the model, with high AUC values obtained for all classes, as shown in Fig. 22. The curves approach the top-left corner, indicating high true-positive rates and low false-positive rates across different classification thresholds.

Overall, these results confirm that the feature-level SMOTE approach achieves robust classification performance, stable training behavior, and strong generalization on unseen data.

### Robustness analysis

To ensure experimental robustness, all models were evaluated across multiple runs using different random seeds. The reported results correspond to the mean and standard deviation, as presented in Table 7. The low standard deviation values indicate that the models are stable and not sensitive to random initialization, confirming the reliability of the proposed approach.

**Table 7:** Performance comparison of transfer learning models using SMOTE (mean ± standard deviation).

Model	Epochs	Accuracy (Mean ± Std)	F1-score (Mean ± Std)	AUC (Mean ± Std)
ShuffleNet	5	0.9713 ± 0.0038	0.9711 ± 0.0039	0.9965 ± 0.0006
GoogLeNet		0.9829 ± 0.0019	0.9828 ± 0.0019	0.9986 ± 0.0002
ShuffleNet	10	0.9823 ± 0.0037	0.9821 ± 0.0037	0.9972 ± 0.0005
GoogLeNet		0.9793 ± 0.0026	0.9792 ± 0.0026	0.9976 ± 0.0003

#### K-fold cross-validation

To further validate the generalization capability of the proposed approach, a threefold cross-validation was conducted using ShuffleNet as a representative model. As shown in Table 8, the results demonstrate consistent performance across different folds, achieving an average accuracy of 97.13% ± 0.38% and an F1-score of 97.11% ± 0.39%. The low standard deviation values confirm that the model is stable and not sensitive to variations in data splits. The cross-validation accuracy (97.13%) is slightly lower than the independent test accuracy (98.35%), which is expected because each fold is evaluated on unseen validation partitions, whereas the final test result corresponds to the best-performing trained model. The relatively small difference further supports the generalization capability and robustness of the proposed framework.

**Table 8:** Performance of ShuffleNet using threefold cross-validation.

Fold	Accuracy	F1-score
1	0.9714	0.9714
2	0.9758	0.9757
3	0.9665	0.9661

#### Statistical significance analysis

To further assess whether the performance difference between the two best-performing models, ShuffleNet and GoogLeNet, was statistically meaningful, a paired t-test was conducted. The test was performed using the fold-wise performance values obtained during model comparison.

As shown in Table 9, the obtained t-statistics were 4.31 for accuracy and 4.30 for F1-score, with 2 degrees of freedom (df = 2). The corresponding p-values were 0.050109 and 0.050153, respectively, which are very close to the commonly adopted significance threshold of 0.05.

**Table 9:** Statistical comparison between ShuffleNet and GoogLeNet using a paired t-test.

Metric	t-statistic	df	*p*-value
Accuracy	4.31	2	0.050109
F1-score	4.30	2	0.050153

These results indicate that although GoogLeNet achieved slightly higher average performance than ShuffleNet, the observed differences are only marginally significant. Therefore, both models can be considered highly effective and reliable for the investigated SEM image classification task, while GoogLeNet provides a slight performance advantage.

To further quantify the uncertainty associated with the reported results, 95% confidence intervals were computed on the independent test set for the best-performing pixel-level and feature-level models. As shown in Table 10, both approaches exhibit relatively narrow confidence intervals, indicating stable performance and supporting the reliability of the reported results despite class imbalance.

**Table 10:** Classification performance with 95% confidence intervals on the test set.

Approach	Accuracy (%)	CI (%)
Pixel-level SMOTE	98.35	97.59–99.11
Feature-level SMOTE	97.44	96.51–98.37

#### Distribution shift analysis of SMOTE samples

To investigate whether the synthetic samples generated by SMOTE introduce a distribution shift relative to the original SEM images, dimensionality reduction techniques were employed to visualize the feature distributions of real and synthetic samples. Specifically, deep feature embeddings were extracted using a pre-trained ResNet50 model and projected into a two-dimensional space using both t-SNE and UMAP.

**Fig. 23:**
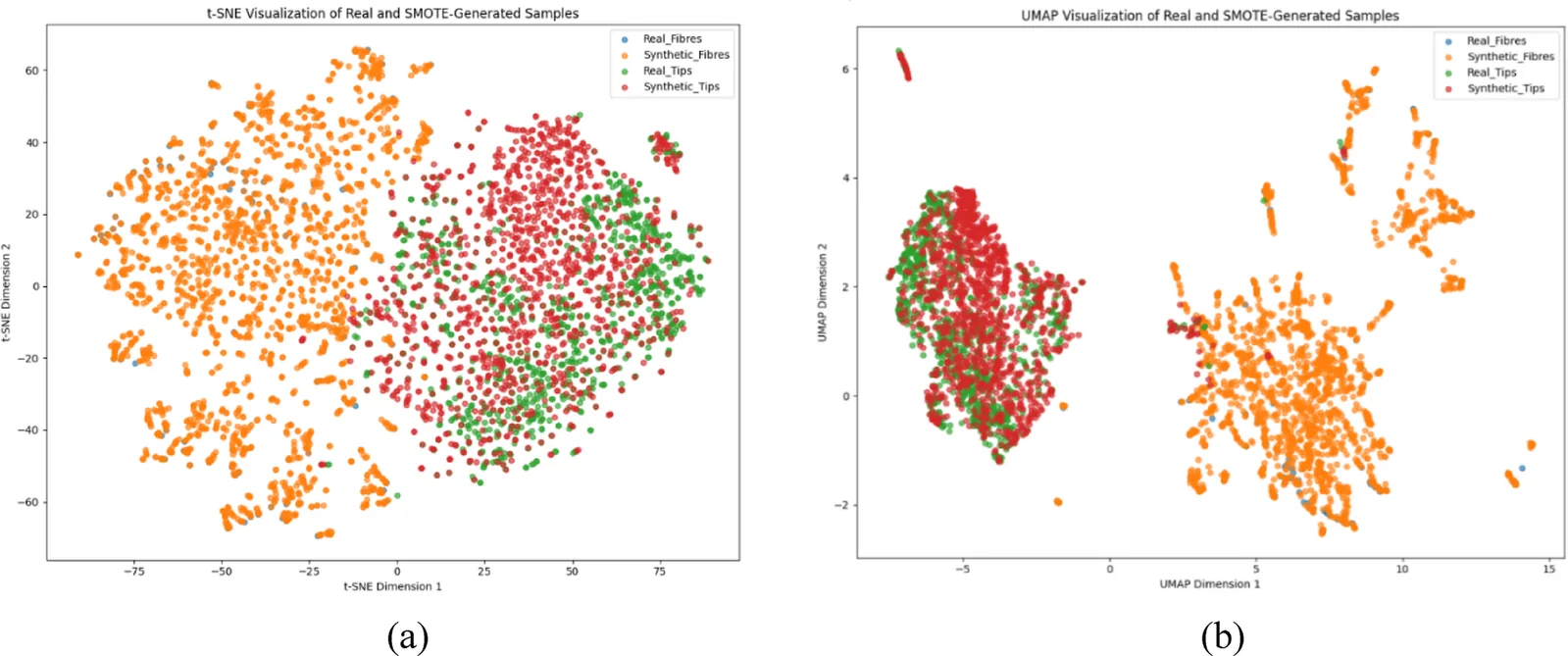
(**a**) t-SNE, (**b**) UMAP visualization of real and SMOTE-generated samples for the Fiber and Tip classes.

Figure 23 illustrate the distributions of real and synthetic samples for the Fiber and Tip classes. The visualizations show substantial overlap between real and synthetic samples within the same class, indicating that the generated samples largely follow the underlying data distribution rather than forming isolated clusters. This suggests that SMOTE generated samples remain consistent with the original feature space and do not introduce significant out-of-distribution artifacts.

Furthermore, the synthetic samples preserve the overall class structure while increasing sample density in minority regions of the feature space. These observations support the use of SMOTE as an effective balancing strategy for SEM image classification and reduce concerns regarding synthetic sample memorization or distribution shift.

### Comparative analysis

This section provides a comparative analysis to evaluate the effectiveness of the proposed approach from multiple perspectives. The evaluation examines the impact of data balancing strategies, feature representations, and model architectures on classification performance, robustness, generalization capability, and computational efficiency in SEM image classification.

#### Impact of SMOTE on CNN models

To evaluate the effectiveness of SMOTE in addressing class imbalance over 20 epochs, the performance of SqueezeNet, ShuffleNet, and GoogLeNet was compared before and after applying SMOTE. The overall findings with key evaluation measures: Accuracy, F1-Score, Precision, Recall, and AUC are summarized in Table 11.

**Table 11:** The performance of the models SqueezeNet, ShuffleNet, and GoogLeNet before and after applying SMOTE technique at 20 epochs.

		Accuracy	F1-Score	Precision	Recall	AUC
SqueezeNet	Imbalanced	96.98	94.05	93.13	95.04	97.95
	After SMOTE	96.89	96.93	97	96.89	99.52
ShuffleNet	Imbalanced	97.8	95.5	95.45	95.56	99.8
	After SMOTE	98.35	98.33	98.34	98.35	99.69
GoogLeNet	Imbalanced	96.89	90.58	89.17	92.15	99.48
	After SMOTE	98.35	98.34	98.34	98.35	99.91

On the imbalanced dataset, ShuffleNet achieved the highest performance, which can be attributed to its lightweight architecture and strong generalization capability under imbalanced conditions. However, after applying SMOTE, all models exhibited notable improvements, particularly in recall and F1-score, indicating better recognition of minority classes.

**Fig. 24:**
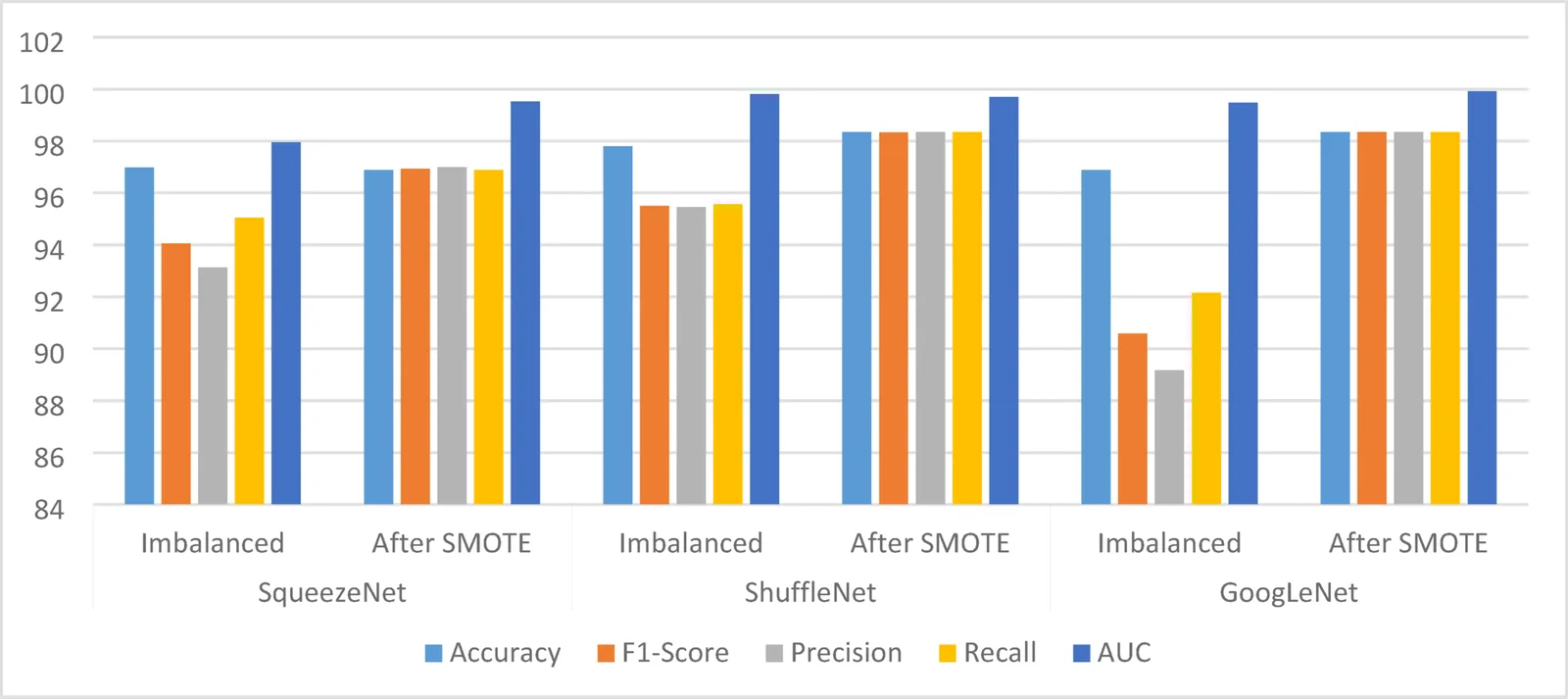
The performance comparison among the three proposed architectures before and after using the SMOTE technique.

Although GoogLeNet achieves the highest AUC, ShuffleNet offers a strong balance between performance and computational efficiency due to its lightweight architecture. These findings demonstrate that SMOTE effectively improves classification performance while maintaining model stability. Performance comparison of SqueezeNet, ShuffleNet, and GoogLeNet before and after applying the SMOTE technique over 20 epochs is shown in Fig. 24.

The above results demonstrate that applying SMOTE improves the classification performance of SqueezeNet, ShuffleNet, and GoogLeNet for NFT classification in SEM images, particularly for the minority classes. The performance of the proposed method and a method that exists^29^ using the same dataset are compared in this section.

#### Pixel-level versus feature-level SMOTE

To further investigate the effect of data representation on model performance, a comparison was conducted between pixel-level SMOTE and feature-level SMOTE approaches. The results are presented in Table 12. The pixel-level approach, when combined with deep CNN architectures such as GoogLeNet and ShuffleNet, achieved the highest performance across all evaluation metrics. This can be attributed to the ability of CNNs to learn spatial and hierarchical features directly from image data in an end-to-end manner.

**Table 12:** Pixel-level versus feature-level SMOTE.

Approach	Models	Accuracy	F1-score	Precision	Recall	AUC
Pixel-level SMOTE	GoogLeNet	98.35	98.34	98.34	98.35	99.91
	ShuffleNet	98.35	98.33	98.34	98.35	99.69
Feature-level SMOTE	MLP (ResNet50)	97.44	97.45	97.52	97.44	99.76

In contrast, the feature-level approach utilized ResNet50 as a fixed feature extractor followed by an MLP classifier. Although this approach resulted in slightly lower performance, it still demonstrated strong classification capability, achieving high accuracy and AUC scores.

These findings indicate a trade-off between performance and computational efficiency. The pixel-level SMOTE approach, integrated within end-to-end deep learning pipelines, yields higher classification performance. However, the feature-level SMOTE approach offers a more efficient solution by eliminating the need for image reconstruction and reducing computational overhead, while still maintaining competitive performance.

#### SMOTE versus data augmentation

To further validate the effectiveness of SMOTE, we compared it with conventional data augmentation techniques for handling class imbalance in SEM image classification. As shown in Table 13, SMOTE consistently outperformed conventional augmentation methods, particularly in terms of F1-score, indicating better handling of class imbalance.

**Table 13:** Comparison between SMOTE and conventional data augmentation techniques for SEM image classification.

Models	Method	Accuracy (%)	Precision (%)	Recall (%)	F1-score (%)	AUC score (%)
SqueezeNet	Data augmentation	81.63	86.74	81.63	80.25	94.63
	SMOTE	96.89	97	96.89	96.93	99.52
ShuffleNet	Data augmentation	93.91	95.32	65	65.42	98.83
	SMOTE	**98.35**	98.34	98.35	98.33	99.69
GoogLeNet	Data augmentation	68.71	80.23	68.71	58.47	98.74
	SMOTE	**98.35**	98.34	98.35	98.33	99.91

Significant values are in [bold].

#### Ablation study of imbalance handling strategies

To further investigate the effectiveness of SMOTE, an ablation study was conducted by comparing four training settings: the original imbalanced dataset (without any balancing technique), random undersampling, class-weighted loss, and SMOTE. All experiments were performed using the ShuffleNet architecture under the same training configuration and for 20 training epochs to ensure a fair comparison. The results are summarized in Table 14.

**Table 14:** Comparison of different imbalance-handling strategies using ShuffleNet.

Method	Accuracy (%)	F1-score (%)	AUC (%)
Imbalanced dataset	97.8	95.5	99.8
Random undersampling	83.53	85.20	96.22
Class-weighted loss	97.8	97.8	99.73
SMOTE	98.35	98.33	99.69

The baseline model trained on the original imbalanced dataset achieved a high overall accuracy of 97.8%; however, its F1-score was noticeably lower (95.5%), indicating that the model was less effective in recognizing minority-class samples despite its strong overall classification accuracy. Random undersampling produced the lowest performance among all evaluated methods. By reducing the majority classes to match the minority class size, the training set was reduced from 3494 samples to only 309 samples, resulting in a substantial loss of information and a significant decrease in classification performance.Class-weighted loss improved minority-class recognition by assigning larger penalties to errors on underrepresented classes without modifying the dataset distribution. This strategy substantially improved the F1-score from 95.5 to 97.8%, demonstrating its effectiveness in mitigating class imbalance.

Nevertheless, SMOTE achieved the best overall classification performance, obtaining 98.35% accuracy and 98.33% F1-score. Unlike class-weighted loss, SMOTE increases minority-class representation through synthetic sample generation while preserving all original training samples. These results indicate that SMOTE provides a more effective balance between class representation and information preservation and achieved the best overall performance among the evaluated imbalance-handling strategies.

#### Comparison with state-of-the-art

To further validate the effectiveness of the proposed approach, a comparative analysis was performed between the three proposed architectures (GoogLeNet, ShuffleNet, and SqueezeNet) using the SMOTE technique and the existing method introduced by Wong^29^. This comparison highlights not only the classification accuracy but also the computational efficiency of the models in handling NFT objects on SEM images.

The results presented in Table 15 clearly demonstrate that the proposed GoogLeNet with SMOTE achieved higher accuracy (98.35%) compared to Wong’s GoogLeNet model (97.1% accuracy with data augmentation). While the training time of GoogLeNet with SMOTE was longer (32 min and 58 s), ShuffleNet achieved the same accuracy (98.35%) with a significantly shorter processing time of only 8 min and 18 s, underscoring its computational efficiency. SqueezeNet, while achieving a slightly lower accuracy (96.89%), maintained competitive results with a moderate training time of 10 min and 23 s.

**Table 15:** A comparison of the performance matrices between the proposed method using SMOTE technique against state of the art model.

Metrics	Wong et al.yy^29^ (with data augmentation)		Proposed method (with SMOTE technique)	
	GoogLeNet	GoogLeNet	ShuffleNet	SqueezeNet
Accuracy	97.1	98.35	98.35	96.89
Time	19 min	32 min 58 s	8 min 18 s	10 min 23 s
F1-Score	Not reported	98.34	98.33	96.93
Precision	Not reported	98.34	98.34	97
Recall	Not reported	98.35	98.35	96.89
AUC	Not reported	99.91	99.69	99.52

It should be noted that these execution times were obtained under the experimental conditions used in this study on Google Colab resources and are reported to provide a relative comparison of computational efficiency among the evaluated models. Due to variations in shared hardware allocation, the exact execution times may differ across runs and computing environments.

**Fig. 25:**
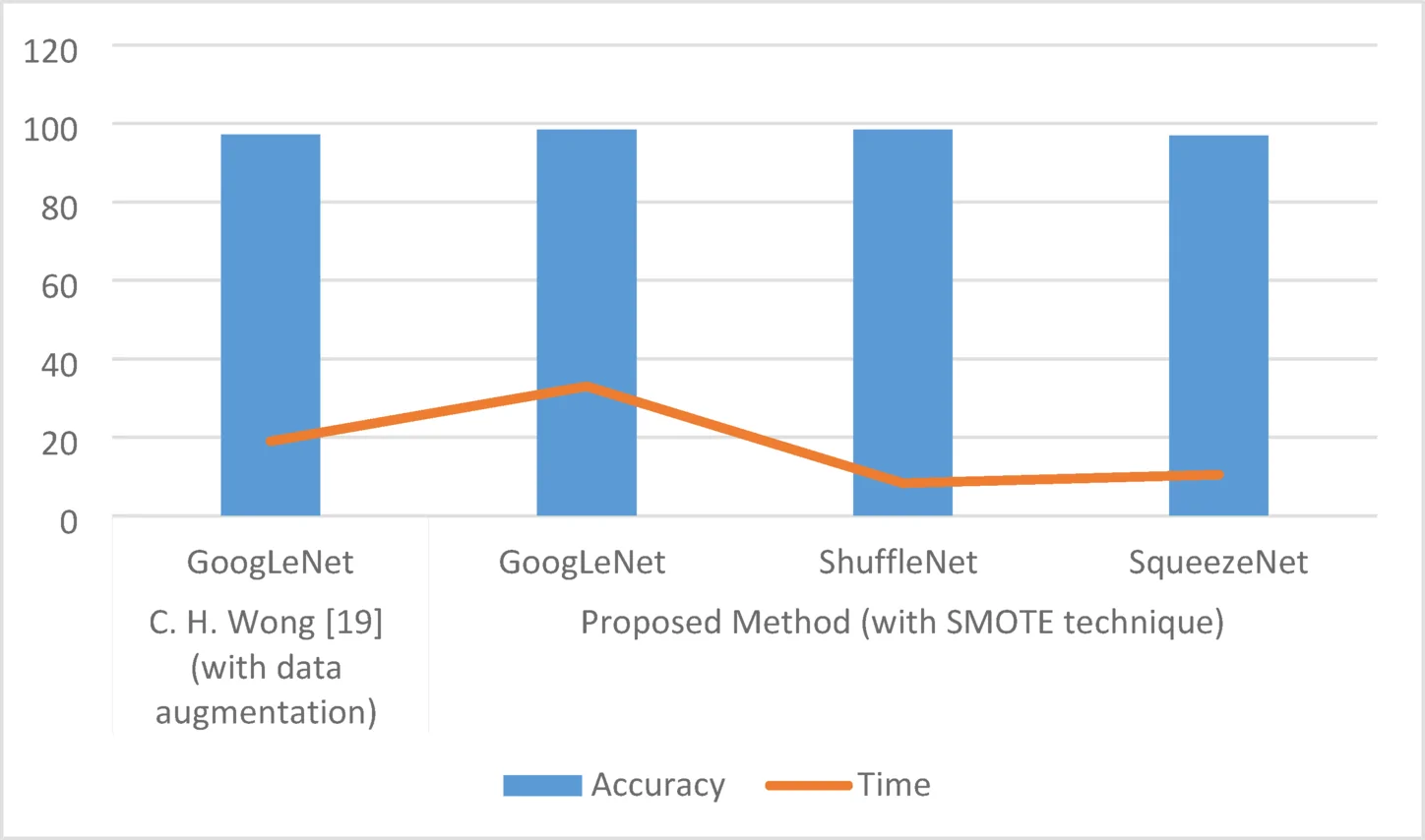
Comparison of state-of-the-art Model^29^ versus Proposed approach using SMOTE.

Overall, the results indicate that applying SMOTE contributes to improved classification performance across the evaluated models. Figure 25 illustrates this comparison, demonstrating that the proposed approach performs favorably compared to the method in Wong et al.^29^ in terms of both accuracy and computational efficiency. It is important to note that this comparison is limited to the selected baseline method and does not represent a comprehensive evaluation against all state-of-the-art approaches.

Notably, the proposed method reports additional metrics not available in the existing method, such as F1-Score, Precision, Recall, and AUC. GoogLeNet achieved the highest AUC of 99.91%, reflecting its superior ability to distinguish between classes. In addition, the proposed method reports a wider range of evaluation metrics, including F1-score, precision, recall, and AUC, which were not provided in Wong et al.^29^. These metrics offer a more comprehensive assessment of model performance.

## Conclusions

This paper presented a comprehensive framework for the classification of nanostructures in SEM images by integrating transfer learning with the Synthetic Minority Over-sampling Technique (SMOTE). To effectively address class imbalance, two complementary SMOTE strategies were investigated: pixel-level and feature-level representations. This dual approach enabled a more thorough analysis of how data representation influences classification performance. Experimental results demonstrated that applying SMOTE significantly improved the classification of minority classes (fibers and tips) while reducing bias toward the majority class (nanowires). Across the evaluated models, both GoogLeNet and ShuffleNet consistently achieved strong and reliable performance. GoogLeNet achieved the highest overall results, with an accuracy of 98.35%, F1-score of 98.34%, and AUC of 99.91%, reflecting its strong capability in capturing complex morphological features. ShuffleNet delivered comparable performance while requiring significantly less computational time, making it a practical alternative for resource-constrained environments. In addition, the feature-level SMOTE approach, combined with an MLP classifier, achieved an accuracy of 97.44%, with an F1-score of 97.45% and an AUC of 99.76%. Despite operating in a lower-dimensional feature space, this approach demonstrated competitive performance while reducing computational complexity by avoiding image reconstruction. This highlights an important trade-off between representation efficiency and model complexity. The robustness of the proposed framework was further supported by consistent results across multiple runs and cross-validation experiments, with low standard deviation values indicating stable model behavior. Overall, the findings confirm that integrating SMOTE with transfer learning provides an effective solution for handling imbalanced SEM datasets and improving multi-class classification performance. Future work will focus on enhancing model generalization through advanced data augmentation and synthetic data generation techniques, as well as investigating lightweight architectures and deployment strategies for resource-constrained environments. These directions may facilitate the future development of practical automated SEM image analysis systems for nanotechnology applications.

## Data Availability

The data presented in this paper is a public dataset obtained from: 10.1038/sdata.2018.172.

## References

[1] Qian, B., Xu, K., Zhang, D., Komarneni, S. & Xue, D. A multiscale perspective on cluster-based layered materials: Design and function optimization toward catalytic and energy storage applications. *InfoMat*10.1002/inf2.12028 (2019). 10.1016/j.fmre.2023.12.003 PMC12327831 40777769

[2] Oganov, A. R., Pickard, C. J., Zhu, Q. & Needs, R. J. Structure prediction drives materials discovery. *Nat. Rev. Mater.* **4**(5), 331–348. 10.1038/s41578-019-0101-8 (2019).

[3] Khan, I., Saeed, K. & Khan, I. Nanoparticles: Properties, applications and toxicities. *Arab. J. Chem.* **12**(7), 908–931. 10.1016/j.arabjc.2017.05.011 (2019).

[4] Joudeh, N. & Linke, D. Nanoparticle classification, physicochemical properties, characterization, and applications: A comprehensive review for biologists. *J. Nanobiotechnol.* **20**, 262. 10.1186/s12951-022-01477-8 (2022). 10.1186/s12951-022-01477-8 PMC9171489 35672712

[5] Fu, Y. et al. Applications of nanomaterial technology in biosensing. *J. Sci. Adv. Mater. Devices* **9**(3), 100694. 10.1016/j.jsamd.2024.100694 (2024).

[6] Datye, A. & DeLaRiva, A. Scanning electron microscopy (SEM). In *Springer Handbook of Advanced Catalyst Characterization* (eds Wachs, I. E. & Bañares, M. A.) 575–598 (Springer, 2023). 10.1007/978-3-031-07125-6_18.

[7] Aziz, A., Shaikh, H., Abbas, A., Zehra, K. E. & Javed, B. Microscopic techniques for nanomaterials characterization. *J. Microsc. Microanal.* **29**(5), 1159–1174. 10.1002/jemt.24799 (2023). 10.1002/jemt.24799 39780429

[8] Wei, J. et al. Machine learning in materials science. *Infomat* **1**(3), 338–358. 10.1002/inf2.12028 (2019).

[9] Li, Q. et al. MD-HIT: Machine learning for material property prediction. *npj Comput. Mater.* **10**, 1426. 10.1038/s41524-024-01426-z (2024).

[10] Liu, Y., Zhao, T., Ju, W. & Shi, S. Materials discovery and design using machine learning. *J. Materiomics* **3**(3), 159–177. 10.1016/j.jmat.2017.08.002 (2017).

[11] Yu, Y. Deep learning approaches for image classification. *Proc. ACM Int. Conf.* **3573428**, 3573691. 10.1145/3573428.3573691 (2022).

[12] Hirabayashi, Y. et al. Deep learning for three-dimensional segmentation of electron microscopy images of complex ceramic materials. *npj Comput. Mater.* **10**, 46. 10.1038/s41524-024-01226-5 (2024).

[13] Lin, B. et al. A deep learned nanowire segmentation model using synthetic data augmentation. *npj Comput. Mater.* **8**, 88. 10.1038/s41524-022-00767-x (2022).

[14] Ge, M., Su, F., Zhao, Z. & Su, D. Deep learning analysis on microscopic imaging in materials science. *Mater. Today Nano* **11**, 100087. 10.1016/j.mtnano.2020.100087 (2020).

[15] Azad, M. M., Prabhakar, M. N. & Kim, H. S. Deep learning-based autonomous morphological fracture analysis of fiber-reinforced composites. *Eng. Fail. Anal.* **170**, 109292. 10.1016/j.engfailanal.2025.109292 (2025).

[16] Perkgoz, C. Identifying optical microscope images of CVD-grown two-dimensional MoS_2_ by convolutional neural networks and transfer learning. *PeerJ Comput. Sci.* **10**, e1885. 10.7717/peerj-cs.1885 (2024). 38435565 10.7717/peerj-cs.1885 PMC10909165

[17] Manujesh, B. J. & Prajna, M. R. Damage detection and classification for sandwich composites using machine learning. *Mater. Today Proc.* **56**, 3159–3164. 10.1016/j.matpr.2021.10.088 (2021).

[18] Dey, B., Goswami, D., Halder, S., Khalil, K., Leray, P. & Bayoumi, M. A. Deep learning-based defect classification and detection in SEM images. arXiv preprint http://arxiv.org/abs/2206.13505. 10.48550/arXiv.2206.13505 (2022).

[19] Jin, Q., Jiang, Y., Lu, X., Liu, Y., Chen, Y., Gao, D., Sun, Q. & Zhuo, C. SEM-CLIP: Precise few-shot learning for nanoscale defect detection in scanning electron microscope images. arXiv preprint http://arxiv.org/abs/2502.14884. 10.48550/arXiv.2502.14884 (2025).

[20] Huang, C.-F. F., Sieg, K., Karlinksy, L., Flores, N., Sheraw, R., & Zhang, X. Semiconductor SEM image defect classification using supervised and semi-supervised learning with vision transformers. In *Proceedings of the ACM International Conference on Defect Inspection and Reduction* 1–8 (ACM, 2025). 10.1145/3676536.3676752.

[21] Ding, X., Zhang, Y., Ge, Y., Zhao, S., Song, L., Yue, X. & Shan, Y. UniRepLKNet: A universal perception large-kernel ConvNet for audio, video, point cloud, time-series and image recognition. arXiv preprint http://arxiv.org/abs/2311.15599. 10.48550/arXiv.2311.15599 (2023).

[22] Srinivas, S. S., Sarkar, R. K., & Runkana, V. EMCNet: Graph-Nets for electron micrographs classification. arXiv preprint http://arxiv.org/abs/2409.03767. 10.48550/arXiv.2409.03767 (2024).

[23] Modarres, M. H. et al. Neural network for nanoscience scanning electron microscope image recognition. *Sci. Rep.* **7**, 13282. 10.1038/s41598-017-13565-z (2017). 29038550 10.1038/s41598-017-13565-z PMC5643492

[24] Kavuran, G. SEM-Net: Deep features selections with binary particle swarm optimization method for classification of scanning electron microscope images. *Mater. Today Commun.* **29**, 102198. 10.1016/j.mtcomm.2021.102198 (2021).

[25] López Gutiérrez, J. D., Abundez Barrera, I. M. & Torres Gómez, N. Nanoparticle detection on SEM images using a neural network and semi-synthetic training data. *Nanomaterials* **12**(11), 1818. 10.3390/nano12111818 (2022). 35683674 10.3390/nano12111818 PMC9182493

[26] Barua, P. D. et al. NFSDense201: Microstructure image classification based on non-fixed size patch division with pre-trained DenseNet201 layers. *Neural Comput. Appl.* **35**, 22253–22263. 10.1007/s00521-023-08825-1 (2023).

[27] Qian, Z., et al. Zero-shot nanoparticle shape classification using vision foundation models. arXiv preprint http://arxiv.org/abs/2508.03235. 10.48550/arXiv.2508.03235 (2025).

[28] Datta, A. et al. Deep learning-based framework for high-throughput nanoparticle analysis in SEM and TEM images. *Nanoscale* **14**(20), 7567–7577. 10.1039/d2nr01029a (2022).

[29] Wong, C. H., Ng, S. M., Leung, C. W. & Zatsepin, A. F. The effectiveness of data augmentation of SEM images on a small database based on deep-learning intelligence. *Braz. J. Phys.*10.1007/s13538-021-01008-0 (2021).

[30] Aversa, R. et al. The first annotated set of scanning electron microscopy images for nanoscience. *Sci. Data* **5**, 180172. 10.1038/sdata.2018.172 (2018). 30152811 10.1038/sdata.2018.172 PMC6111892

[31] Chen, W. et al. A survey on imbalanced learning: Latest research, applications and future directions. *Artif. Intell. Rev.* **57**, 137. 10.1007/s10462-024-10759-6 (2024).

[32] Elreedy, D. & Atiya, A. F. A comprehensive analysis of synthetic minority oversampling technique (SMOTE) for handling class imbalance. *Inf. Sci.* **505**, 32–64. 10.1016/j.ins.2019.07.070 (2019).

[33] Chamseddine, E., Mansouri, N., Soui, M. & Abed, M. Handling class imbalance in COVID-19 chest X-ray images classification: Using SMOTE and weighted loss. 10.1016/j.asoc.2022.109588 (2022). 10.1016/j.asoc.2022.109588 PMC9422401 36061418

[34] Yosinski, J., Clune, J., Bengio, Y. & Lipson, H. How transferable are features in deep neural networks? arXiv preprint http://arxiv.org/abs/1411.1792. 10.48550/arXiv.1411.1792 (2014).

[35] Zhang, X., Zhou, X., Lin, M. & Sun, J. ShuffleNet: An extremely efficient convolutional neural network for mobile devices. In *Proceedings of the IEEE Conference on Computer Vision and Pattern Recognition (CVPR)*, 6848–6856. 10.48550/arXiv.1411.1792 (2018).

[36] Iandola, F. N., Han, S., Moskewicz, M. W., Ashraf, K., Dally, W. J. & Keutzer, K. SqueezeNet: AlexNet-level accuracy with 50x fewer parameters and < 0.5 MB model size. arXiv preprint http://arxiv.org/abs/1602.07360. 10.48550/arXiv.1602.07360 (2016).

[37] Szegedy, C., Liu, W., Jia, Y., Sermanet, P., Reed, S., Anguelov, D., et al. Going deeper with convolutions. In *Proceedings of the IEEE Conference on Computer Vision and Pattern Recognition (CVPR)*, 1–9. 10.1109/CVPR.2015.7298594 (2015).

